# Longitudinal tracking of healthcare professionals: a methodological scoping review

**DOI:** 10.1186/s12874-025-02533-1

**Published:** 2025-04-01

**Authors:** Yingxi Zhao, Xuan Li, Attakrit Leckcivilize, Mike English

**Affiliations:** 1 Nuffield Department of Medicine, NDM Centre for Global Health Research, University of Oxford, S Parks Rd, Oxford, OX1 3SY UK; 2 Nuffield Department of Population Health, University of Oxford, Oxford, UK; 3 KEMRI-Wellcome Trust Research Programme, Nairobi, Kenya

**Keywords:** Human resources for health, Tracking, Longitudinal study, Cohort, Workforce

## Abstract

**Background:**

Tracking and understanding the progress and experiences of health workers and the outcomes of workforce decisions are essential for evidence-based workforce planning. In this scoping review, we aim to identify longitudinal studies that prospectively tracked healthcare professionals and that specifically focused on workforce issues such as career preferences, choices, and working conditions, and summarise the different approaches and methods used for tracking.

**Methods:**

We searched MEDLINE, Embase, Global Health, PsycINFO, CINAHL, Education Resource Information Center (ERIC), EconLit and the Cochrane Library for articles published between 2000–2022 that longitudinally tracked doctors, nurses, midwives, physician associates/assistants. We further compared articles and conducted a back-and-forward citation search to identify longitudinal tracking *studies* which sometimes have multiple published *articles*. We developed a typology of the different tracking approaches, and summarised the major areas assessed and tracked by different studies.

**Results:**

We identified and analysed 263 longitudinal tracking studies. Based on population recruitment and follow-up methods, we grouped studies into seven categories (cohort studies, multiple-cohort studies, baseline and data linkage studies, baseline and short repeated measure studies, baseline-only studies, data linkage-only studies and repeated survey studies). The majority of studies included used a cohort or multiple-cohort design (*n* = 180), and several others also used data linkage (n = 45) and repeated measure approaches (*n *= 24). Sixty-two studies recruited participants while they were students and followed them until they became the active workforce, and nearly half of the included studies started directly from the active workforce stage. Most of the included studies examined workforce issues including employment status, preference or intention (to leave/remain/migrate, specific speciality or location etc.), and work environment, however there was a lack of widely used measurement tools for workforce issues. Additionally, nearly 40% examined wellbeing issues and a subset (20%) examined physical health in the context of workforce-related issues.

**Conclusion:**

We described a large number of different healthcare professional longitudinal tracking studies. In order for longitudinal tracking to contribute to effective workforce planning, we recommend employing a mix of cohort and data linkage approaches to collect data across the different stages of the workforce ‘working lifespan’, and using and continuing to test standardised measurement instruments to better capture experiences related to workforce and wellbeing.

**Supplementary Information:**

The online version contains supplementary material available at 10.1186/s12874-025-02533-1.

## Background

Health systems can only function with health workers [1]. The World Health Organization (WHO) projected that globally there were 65.1 million health workers in 2020 and that an additional 10 million health workers are needed by 2030 [2]. Additionally, many countries face challenges in the maldistribution of healthcare professionals especially in rural and remote areas. The Covid-19 pandemic further highlighted challenges related to healthcare workers’ wellbeing and morale, which negatively impacted workforce productivity and service delivery [3].

Tracking and understanding the progress and experiences of health workers, as well as the outcomes of workforce decisions are essential for planning. While many governments and regulatory authorities collect workforce data through censuses or administrative records, data are mostly cross-sectional and limited to understanding how many healthcare professionals are available and where they are distributed [4]. Most data are not linked over time at the individual level and this limits analyses and the range of information available [5].

Longitudinal tracking of healthcare professionals, where individuals are tracked on career preferences, job satisfaction, work location and environment over time could help researchers, medical educators, human resource managers and policymakers make better evidence-based workforce plans [6, 7]. It could also be used to proactively design and test the effects of policies and interventions to address a range of workforce challenges, such as attracting and retaining healthcare professionals in highly needed specialities, tackling workforce shortages in rural and remote areas, or changes in workforce participation and working hours arrangements to improve productivity and efficiency [6–9]. As an example, longitudinal studies tracking doctors in the UK have been used to widen medical school access and enhance primary care recruitment [10].

There is a wide range of approaches for tracking healthcare professionals longitudinally, yet no comprehensive summary or typology currently exists to guide researchers and policymakers in selecting the most effective tracking methods. In this scoping review, we aim to fill this gap and map the range of longitudinal studies that have prospectively tracked healthcare professionals and that specifically focused on workforce issues. Rather than focusing on specific study details, we summarise the different approaches and methods used, as well as the major areas assessed and tracked. While our findings do not directly inform specific workforce outcomes, we hope they can assist researchers, medical regulators, educators, and policymakers in selecting and designing the most appropriate tracking methods, which could ultimately contribute to effective workforce planning.

## Methods

We conducted this methodological scoping review to identify studies that longitudinally tracked healthcare professionals, specifically doctors, nurses, midwives, and physician associates/assistants, as these represent some of the largest workforce groups. We recognise, however, that other allied health professionals are also crucial to workforce planning. We focused on the different approaches for tracking and the major areas assessed and tracked. Our methodological review is informed by relevant guidance [11–13].

### Search strategy and screening

In consultation with an experienced librarian, we conducted a systematic search using MEDLINE, Embase, Global Health, PsycINFO, CINAHL, Education Resource Information Center (ERIC), EconLit and the Cochrane Library to obtain relevant research articles. We included articles published between 2000–2022 in English only due to time and resource constraints. We combined keyword terms and phrases related to cohorts, healthcare professionals and workforce (see additional file 1 for an example search strategy). We are interested in workforce-related outcomes including but not limited to career preferences and choices, employment, salaries and working conditions, and migration. We also included articles that focus on health workforce psychological wellbeing such as burnout since they are workforce-related issues. However, we excluded articles that did not report on any workforce or wellbeing questions and only focused on physical health such as monitoring health workers’ Covid-19 infection, or only used healthcare professionals as occupational cohorts to study patterns of health and disease [14]. This was to ensure that the studies we included were directly relevant to the workforce challenges and wellbeing concerns central to our research objectives. Lastly, we excluded studies that are solely retrospective cohorts as they are less relevant for workforce planning.

After deduplication, we imported the citations into Abstrackr for initial title and abstract screening [15]. YZ reviewed all the titles and abstracts to assess eligibility for full-text review, and a random subset of 20% was reviewed by XL. Full texts were reviewed by both YZ and XL to determine inclusion. We resolved disagreements on inclusion at the title and abstract stage or at full-text stage through discussion between the two reviewers.

### Identifying longitudinal tracking studies

Our unit of analysis is longitudinal tracking *studies* which sometimes have multiple published *articles*, for each article we conducted an internal comparison to merge and combine articles into individual studies. This included manually comparing authors, country of study, baseline year of first cohort and baseline sample size. We also conducted a back-and-forward citation search in Google Scholar to identify the oldest and most recent published articles and, where possible, the original study protocol.

### Data extraction and collation

YZ extracted data from included studies and entered them into a Microsoft Excel spreadsheet. The following data items were extracted: name of the study, study design, country or territory of study, study population, rounds of ‘cohort baseline’ and ‘follow-up’, baseline year, baseline recruitment platform and size, first and last follow-up year, follow-up platform and size, most recent available follow-up year and size, follow-up incentives, data linkage strategy, funding source, key areas examined and any standardised scale or questionnaire used. We specifically looked into the methods, results sections, appendices as well as publicly available protocols and survey questionnaires to identify relevant information. Several studies included multiple baseline cohorts: for those, we specifically focused on the first initial cohort to understand their survey methodology, such as follow-up strategy and incentives. Many studies’ survey questionnaires evolved throughout the project and differed between each cohort / each follow-up, we tried to incorporate all available versions when extracting the key areas examined and the scale used.

### Summarising and reporting findings

The first aim of this review is to develop a typology of the different approaches and methods used for longitudinal tracking. Based on how study populations are recruited and followed up, we grouped studies into seven categories:

1. Cohort studies (single baseline and subsequent follow-up)
2. Multiple-cohort studies (multiple baseline and follow-ups)
3. Baseline and data linkage studies (no follow-up survey but longitudinal data derived from other datasets)
4. Baseline and short repeated measure studies (same survey tool used multiple times in a relatively short period)
5. Baseline only studies (claim to be cohort studies but only baseline data available)
6. Data linkage only studies (no baseline and follow-up survey but all data derived by linking different datasets)
7. Repeated survey studies (we only included studies that could link up individuals between different rounds of survey even if linkage was limited because of individuals entering and exiting between surveys)

We paid specific attention to cohort and data linkage categories (category 1, 2, 3 and 6) as they offer the most flexible and relevant approach for examining workforce outcomes of interest. However, we acknowledge that other designs may be more suitable for specific research questions. For example, baseline and short repeated measure studies may be particularly useful for assessing mental and physical health, as they focus on capturing data at multiple points over a short timeframe. We summarised the study characteristics, common strategies for recruitment, follow-up and linkage where relevant. We present selected examples in different countries with different study populations. The full list of studies is presented in additional file 2.

Another aim of this review is to identify and summarise the major themes as a way of categorising the key areas covered by different longitudinal studies and tools. Major themes assessed by different studies gradually emerged during protocol development and title/abstract screening stages, and were refined and finalised iteratively at full-text screening and data extraction stages. We converged on three themes after repeated discussions among study team members: workforce, wellbeing, and physical health. It’s important to note that while physical health was considered, we focused primarily on studies where physical health was examined alongside workforce and wellbeing outcomes, as these were more aligned with our research objectives. Within each theme we also iteratively developed subthemes, for example under workforce we included employment status (current location, specialty, role, contract, etc.), employment preference, training experience, work environment, job satisfaction, workload and work hours, sick leave and absenteeism, and others. To deal with possible overlaps among different (sub)themes that are somewhat subjective, for example, placing job strain under workforce and job stress under wellbeing, we repeatedly discussed subthemes within the study team.

## Results

### Search results and study overview

**Fig. 1:**
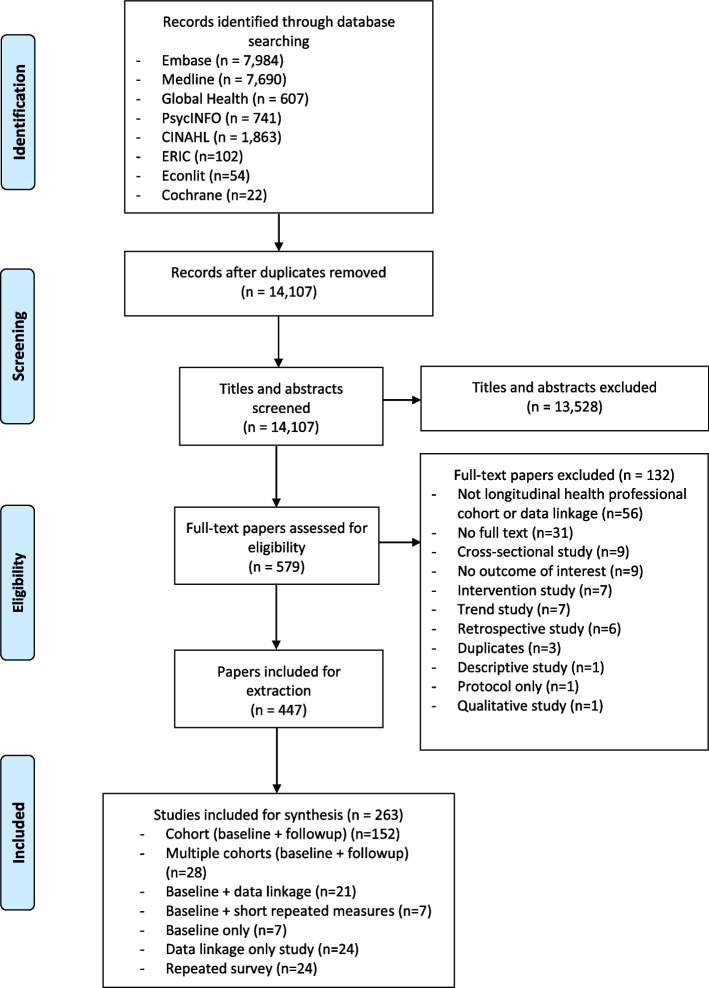
Preferred Reporting Items for Systematic Reviews and Meta‐Analyses (PRISMA) flowchart

**Fig. 2:**
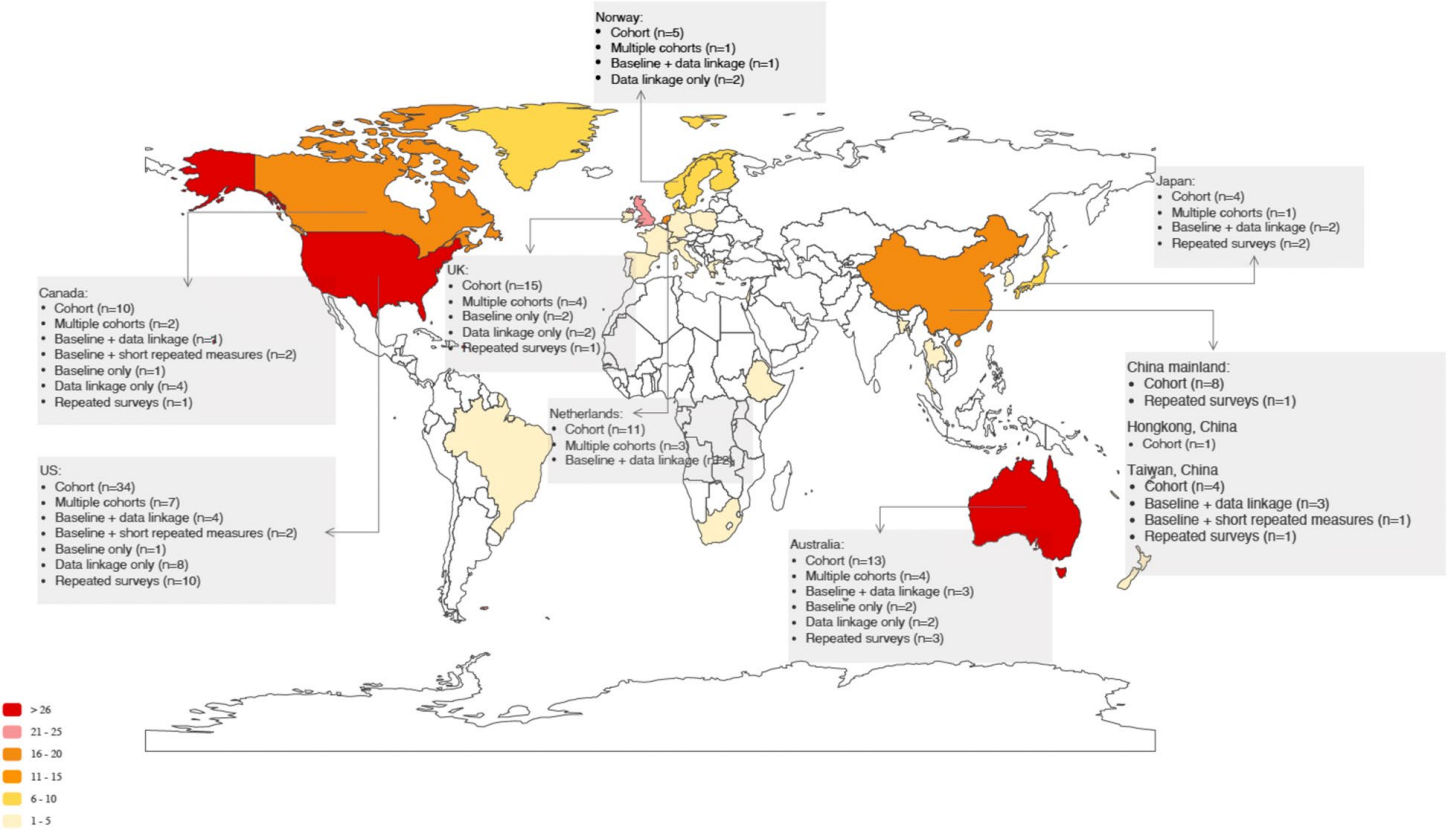
Geographical distributions of different studies. Note: Additional six studies were multi-country studies and not represented in the figure

Figure 1 presents the review process. Of the 14,107 articles identified after deduplications, 447 met the inclusion criteria after full-text screening, resulting in 263 independent studies. As shown in Table 1, the majority of studies were cohort studies (*n* = 152), followed by 28 multiple-cohort studies, 21 baseline and data linkage studies, and 24 studies that relied entirely on data linkage. 42% of studies tracked specifically medical professionals (including different stages from medical student, intern, resident to qualified professionals), while 44% tracked nursing and midwifery professionals. Only one study, the American Academy of PAs (AAPA) student and census survey [16], tracked physician associates. Aside from six multi-country studies, the remaining studies covered 29 countries and territories (Fig. 2). The majority of these studies were conducted in high-income countries and territories, such as the US (*n *= 67), Australia (*n *= 27), UK (*n* = 24), and Canada (*n* = 20), while 18 studies (7%) were conducted in low- and middle-income countries, including Bangladesh, Brazil, China, Ethiopia, South Africa, Thailand.

**Table 1:** Typology and different categories of healthcare professional longitudinal tracking with selected examples

Category	No. of studies	Description	Example studies	Example studies detail
**Cohort studies**	152	• Studies where one single cohort of healthcare professionals were surveyed at baseline and then at follow-up • As for rounds of follow-up, most studies only followed up once (*n* = 86), twice (*n* = 30), or three times (*n* = 17) • Recruitment from training institutions (*n* = 70) and health facilities (*n* = 59) were most common, and 17 studies recruited from professional associations or registries	Survey of Shift work, Sleep and Health (SUSSH) [17, 18]	• Tracked qualified Norway nurses from professional associations (*n* = 2059) • Study started in 2008, 11 rounds of follow-up, expected to still be ongoing
			Nurses' Early Exit Study (NEXT) [19, 20]	• Tracked qualified nurses from 10 countries, from nationally-representative randomly sampled health facilities (*n* = 77,681) • Study started in 2002, 1 round of follow-up
			Wits longitudinal Study to Determine the Operation of the labour Market among its health professional graduates (WiSDOM) [21, 22]	• Tracked South Africa healthcare professional students until after qualification from one training institution (*n* = 457) • Study started in 2017, 3 rounds of follow-up, expected to still be ongoing
**Multiple-cohort studies**	28	• Studies where multiple cohorts of healthcare professionals were surveyed at baseline and then at follow-up • Number of cohorts ranged from 2 cohorts (*n* = 9), 3 cohorts (*n* = 5) to 18 cohorts (*n *= 1) • The justification for a multiple cohort is often to measure and compare period and cohort effects especially related to the policy changes • As for rounds of follow-up for the initial cohort, similarly most studies followed up once (n = 9), twice (*n* = 4), three or four times (*n* = 8) • Recruitment from training institutions (*n* = 19) was most common, followed by professional associations or registries (*n* = 5)	UK Medical Careers Research Group study [23, 24]	• Tracked 16 cohorts of UK medical students until after qualification from professional registry (*n* = 2347) • Study started in 1975, 8 rounds of follow-up
			Longitudinal Analysis of Nursing Education (LANE) [25, 26]	• Tracked 3 cohorts of Sweden nursing students until after qualification from all training institutions (first cohort *n* = 4316) • Study started in 2002, 4 rounds of follow-up, expected to still be ongoing
			Thai Nurse Cohort Study [27, 28]	• Tracked 2 cohorts of qualified Thailand nurse from professional registry (*n* = 18,756) • Study started in 2009, 1 round of follow-up, expected to still be ongoing
**Baseline and data linkage studies**	21	• Studies where commonly the research team conducted a baseline survey and then prospectively linked individuals with registry or administrative records to track workforce data • Most studies (*n* = 20) only had one cohort • Most studies recruited their participants from training institutions (*n* = 8) and health facilities (*n* = 11)	Australia Nursing and Allied Health Graduate Outcomes Tracking (NAHGOT) [29, 30]	• Tracked Australian nurse, midwife, allied health professional students from 3 training institutions (*n* = 1130) • Study started in 2017, linked to regulators’ database
			Finnish Hospital Personnel [31, 32]	• Tracked qualified Finnish doctor and nurse from 11 health facilities (*n* = 930) • Study started in 1997, linked to hospital employee register
**Data linkage studies**	24	• Studies where no prospective data collection was done and all data were collected through linking existing data • The datasets linked varied from training institution databases, professional registries, hospital employer records, to disease registries	UK medical education database (UKMED) [33, 34]	• Tracked UK medical school applicants, students and qualified doctors, started with all entrants to medical schools in 2007 and 2008 (*n* = 15,627) • Study started in 2015, linking training institution and regulators’ database
**Baseline only studies**	7	• Studies that mentioned they were baseline of prospective cohort studies, but we were unable to identify any follow-up articles, which could be due to the studies still conducting follow-up, or the study failed to continue or publish their follow-up results	COVID-19 HEalth caRe wOrkErS [HEROES] study [35]	• Tracked healthcare workers from 26 countries (baseline *n* = 340,000) • Study started in 2020, capturing Covid-19 related exposure and stress
**Baseline and short repeated measure studies**	***7***	• Studies that surveyed participants at baseline and then followed up them very shortly and frequently using one standardised tool such as menstrual cycle diary [36], physical activity tracker [37], or other standardised wellbeing tools • Most studies recruited their participants from health facilities (*n* = 5) or training institutions (*n* = 1)	Resident Activity Tracker Evaluation [37]	• Tracked Canadian medical residents from one health facility (*n* = 59) • Study in 2016 • Residents were first completing a questionnaire on work schedule, physical health and mental health and then asked to wear a FitBit tracker for 14 days to measure sleep and physical activity
**Repeated survey studies**	***24***	• Commonly census or annual surveys, however able to link up individuals throughout different rounds of survey • Most studies recruited their participants from health facilities (*n* = 10) or training institutions (*n* = 6)	American Academy of PAs (AAPA) student and census survey [16]	• Tracked US physician assistants through its professional association (first year *n* = 3721) • Study started in 2000, focusing on specialty and career

We here presented selected examples that focused on different cadres in different countries. The full list of all studies is provided in Additional file 2

### Cohort and multiple-cohort studies

Methodological details of these studies are also presented in additional file 3. Out of 180 cohort and multiple-cohort studies, 152 studies were cohort studies, and 28 studies were multiple-cohorts. Based on the latest publications and publicly available websites, 24 out of these 180 cohort or multiple-cohort studies are expected to be still ongoing.

At baseline, recruiting from training institutions, health facilities, professional associations, professional registries or trade unions was most common. Government authorities and social media were used as recruitment platforms in four studies. For example the Longitudinal Analysis of Nursing Education (LANE) study recruited their participants directly from the national registry by Statistics Sweden [25]. Baseline recruitment in 28 studies aimed to achieve national representativeness of the respective population, although this intention was not explicitly stated in all cases. Around one-third of the studies did not report on their baseline and follow-up survey administration platform, and for studies that did report on this paper-based or mail-based surveys are still the most common.

Retention of participants is an important consideration in cohort studies. The majority of studies (*n* = 140) did not report their strategies for follow-up retention. Where reported commonly used retention strategies included financial incentives (*n* = 12) such as gift cards or lottery draws, or sending reminders (*n* = 19). We compared the retention rates between baseline and the most recently available follow-up data in supplementary Additional file 3. Retention rates are relatively high, with over 50% in most cohort studies, though variations between studies were observed. Those with retention strategies did not necessarily have higher retention rates.

Another consideration for cohort studies is the linkage between different rounds of surveys. While 147 studies did not explicitly report on their linkage approach, 23 studies mentioned the use of a unique identifier, three studies used participants’ names, and other approaches included using a national identification number, social security number, student identification number, or a mix of birth dates and postcodes.

**Fig. 3:**
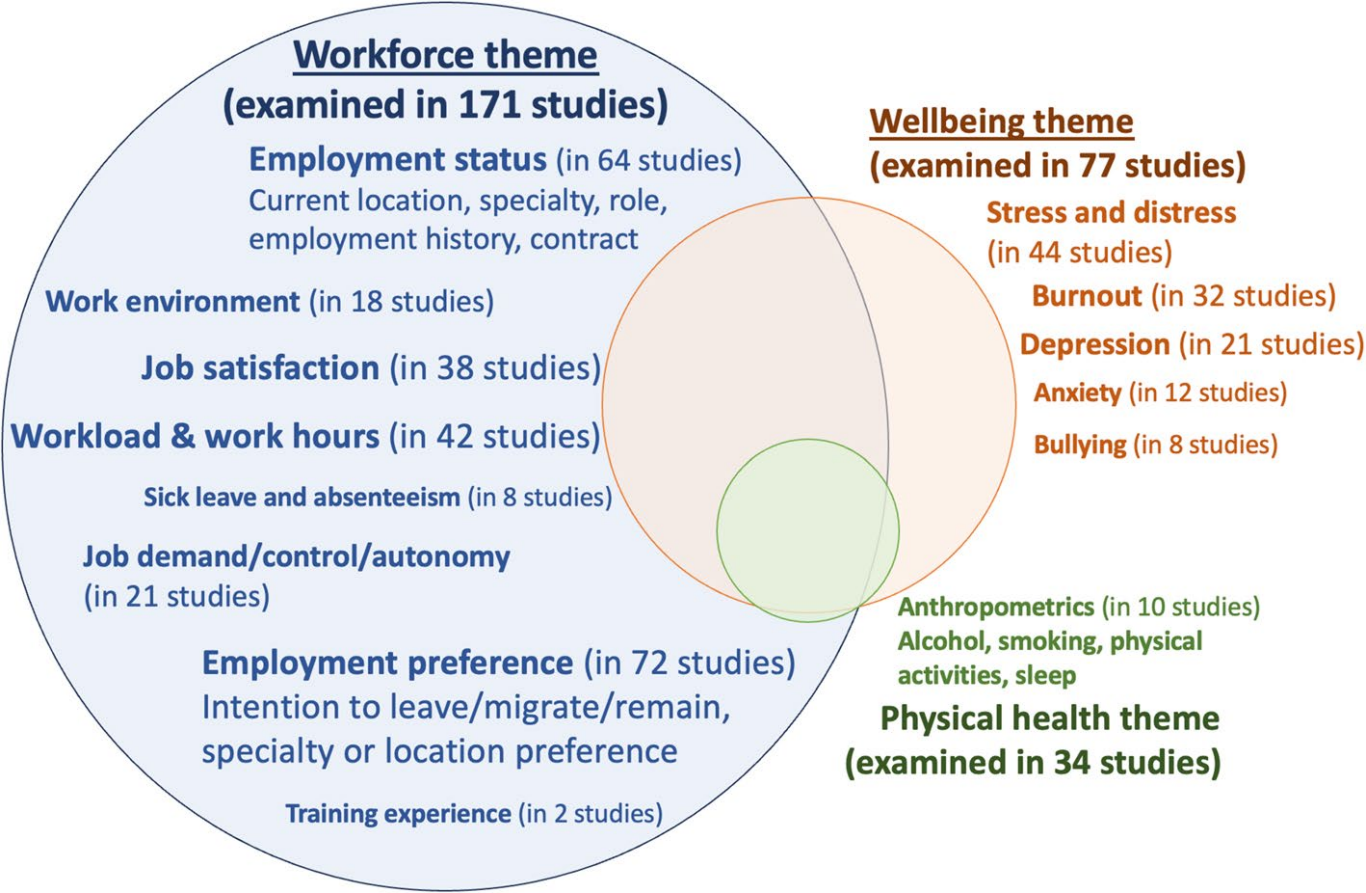
Commonly examined areas by different longitudinal tracking studies. Note: The Venn diagram shows the different areas examined by different studies, categorised into three themes (workforce, wellbeing and physical health). The overlap between the three circles indicates the relative proportion of studies that examined two or more themes. For example 24 studies examined all three themes, and 54 studies examined two themes

In terms of the major themes and areas examined by these cohort and multiple cohort studies, 171, 77, and 34 studies examined topics classified as in our themes of workforce, wellbeing and physical health, respectively. Many articles studied subjects with more than one theme. Figure 3 illustrates the different areas examined in these studies and the overlaps of themes between different studies. While questions for some areas were commonly designed ad hoc (e.g., current employment location or speciality), several measurement tools or scales were used to examine workforce topics, with wellbeing more commonly studied. These included the Job Content Questionnaire (used in 10 studies), the Copenhagen Psychosocial Questionnaire (in 7 studies), the Maslach Burnout Inventory (MBI) (in 19 studies), the Patient Health Questionnaire (PHQ-9) (in 7 studies), and the General Health Questionnaire (GHQ) (in 7 studies) (see Table 2).

**Table 2:** Commonly used measurement tools for workforce, wellbeing and physical health from the scoping review

Commonly used tools	Number of times used	Publicly available link	Number of items	Language version publicly available	Free for academic use
Copenhagen psychosocial questionnaire (COPSOQ)	7	https://www.copsoq-network.org/	32 “core” items, 28 additional “middle” items and 86 additional “long” items for version III	25	Free
Job content questionnaire (JCQ)	10	https://www.jcqcenter.com/	22 (core), 49 (long)	29	Not free
Effort—reward imbalance scale	3	N/A	16 (short), 22 (long)	N/A	Free
Utrecht Work Engagement Scale	3	https://www.wilmarschaufeli.nl/tests/	9 (short), 17 (long)	31	Free
Maslach Burnout Inventory (MBI)	19	https://www.mindgarden.com/117-maslach-burnout-inventory-mbi	22	53	Not free
General Health Questionnaire (GHQ)	7	N/A	12, 28, 30 or 60	N/A	Free
Perceived Stress Scale (PSS)	4	N/A	10	N/A	Free
Patient Health Questionnaire (PHQ-9)	7	N/A	9	N/A	Free
Generalized Anxiety Disorder 7-item (GAD-7)	3	N/A	7	N/A	Free
Center for Epidemiological Studies-Depression (CES-D)	6	N/A	20	N/A	Free
State Trait Anxiety Inventory	4	N/A	20	12	Free
Oldenburg burnout inventory	3	N/A	16	N/A	Free
Short form health survey	4	https://www.rand.org/health-care/surveys_tools/mos/36-item-short-form.html	12, 20 or 36	2	Free

Many of these tools have unofficial but validated translations in published literature. The “language version publicly available” listed here refers to the versions available on related websites

### Baseline and data linkage studies

Twenty-two studies were baseline studies with data linkage. Aside from Arora V et al.’s. US doctor study [38] that had three baseline cohorts at different time points, most other studies only had one cohort. Similar to cohort and multiple-cohort studies, recruitment from training institutions and health facilities were most common.

In terms of data linkage, hospital employers’ records were the database most commonly linked database (*n* = 10), followed by professional registries (*n* = 6), and training-related databases (*n* = 2). One exception is the DAK-Gesundheit nurse study [39] that recruited nurses at baseline from one of the health insurance companies, and linked their baseline responses with the insurance claim record to identify sick leave events. Only four studies reported their data linkage strategies, all of which used a unique identification number.

Regarding the major themes and areas examined, all studies examined workforce topics, seven examined wellbeing and three examined physical health. The major subthemes and measurement tools used for this category of studies are similar to cohort and multiple cohort studies.

### Data linkage only studies

Twenty-four studies used a data linkage design. The datasets linked varied from training institution databases, professional registries, hospital employer records, to disease registries (for example Cancer Registry of Norway in Lie et al.’s Norway nurse study [40]). One specific example of data linkage for education and workforce planning purposes is the UK medical education database (UKMED) where secondary data from pre-medical school throughout postgraduate training and employment are pooled to understand how medics move through education and career. The datasets linked included medical school pre-admission tests (e.g. Clinical Aptitude Test [UKCAT]), medical school training and postgraduate training (e.g. General Medical Council [GMC] registry and national trainee survey census), and practice history data (e.g. from payroll data provided to the GMC for revalidation purpose) [33, 34].

### Other types of longitudinal tracking studies

Thirty-eight studies used other types of study design, including 7 studies that only had baseline data, 7 studies that conducted baseline survey and followed-up with short repeated measures, and 24 studies that we categorised as repeated survey. More detail of these categories is shown in Table 1.

## Discussion

Through reviewing and synthesising research articles published from 263 healthcare professional longitudinal tracking studies, we summarised different approaches and methods used for tracking as well as the major areas assessed and tracked. The majority of studies included used a cohort or multiple-cohort design (*n* = 180), and several others also used data linkage and repeated measure approaches. Most of the included studies examined workforce issues including employment status, preference or intention (to leave/remain/migrate, specific speciality or location etc.), and work environment, nearly 40% examined wellbeing issues and 20% examined physical health.

Sustaining a large cohort study requires a long-term financial investment and in staff and infrastructure to manage survey administration. We found that a large proportion of cohorts used mail- or paper-based surveys, likely due to the fact that many of the studies were conducted in the 1990s and early 2000s. More recent cohorts have used a mix of paper-based and web-based surveys, or completely web-based surveys as they are cost-saving and improve quality control, while smartphones, emails and social media provide more opportunities for identifying, recruiting and following up participants [5, 41, 42]. We also found that the majority of studies did not mention their retention strategies, and those that employed a retention strategy such as gift cards or reminders did not necessarily have a higher retention rate. This is in accord with the literature suggesting that employing a large number of retention strategies might not be associated with improved retention in longitudinal cohort studies, and barrier-reduction such as having a shorter questionnaire, instead of sending out constant follow-up reminders, should be prioritised [43].

The other commonly used alternative or supplement to cohort follow-up is using data linkage. In many countries, the digitalisation of administrative records such as staffing and payroll data, regulatory council and professional association membership facilitated use of these data for workforce planning and analysis [44]. Our findings suggest that training institution databases, professional licensure registries, and hospital employer records were commonly linked databases for healthcare professional longitudinal tracking. According to these data linkage studies, these administrative databases often offer the advantage of being routinely or continuously updated, cost efficiencies, reduced burden on participants, and in theory higher data accuracy and less bias than self-reported data in questionnaire surveys [29, 31]. However, secondary data sources have their own limitations: administrative datasets are usually fragmented, and linking between different sources not only requires time and resources for quality control and tackling linkage errors, but also needs to consider different governance issues such as consent, confidentiality and data protection [45, 46]; also secondary data often only describe *what* happened and rarely *how* and *why* [33]. In many LMICs, government and regulatory bodies still lack the human, financial, infrastructural and technical resources to collect, compile and analyse workforce data [44], as well as legal mandates to ensure data are accurate and up-to-date. For example, a survey of 12 African countries’ medical councils suggested that only two councils collected information on doctors’ current employment sector and many lack the enforcement mechanism to ensure doctors annually renew their licenses [7]. However, progressive strengthening of health workforce information systems and linkage across surveys and other data sources could greatly contribute to workforce planning and decision-making.

The included studies focused on different cadres of healthcare professionals as well as different stages of the ‘working lifespan’. The WHO recommends monitoring the workforce at three key junctures, i.e. when people enter the workforce (considering planning, education and recruitment), when they are an active part of the workforce (issues related to supervision, compensation, system support and lifelong learning), and when they exit (migration, career choices, health and safety, and retirement) [44]. While nearly half of the included studies started from the active workforce stage, commonly recruiting participants from health facilities or professional registries, 62 studies recruited their participants while they were students (at the entry stage) and followed them until they became the active workforce. Tracking healthcare professionals while they are still students provides a more comprehensive picture of the workforce continuum, allows to capture individuals who dropped out before entering the workforce, and may inform design and testing of interventions and policies to address educational challenges. However, coordinating data collection activities between different training institutions could be challenging in countries where there are a large number of medical and nursing schools, especially if the aim is to produce nationally representative data to assist workforce planning. In those cases, retrospectively linkage to training institution databases could be an option.

It's worth emphasising that the use of longitudinal tracking of healthcare professionals is scarce in LMICs, as only 7% of the included studies are conducted in LMICs. It is unfortunate that countries most in need of workforce strengthening often have the least – or most fragmented and unreliable data to track their workforce status [44]. While this could be due to limitations in human, financial, infrastructural and technical resources as well as legal mechanisms discussed above, we did identify a few exemplars that others could learn from. The Thai Nurse Cohort Study started in 2007, sampling 18,756 nurses from its professional registry, and has conducted at least two rounds of follow-up with an over 60% retention rate as responses were legally required based on continuing education credits and professional registry renewal. It not only served as a workforce cohort to help address the Thai nursing workforce crisis but also contributed as a physical health cohort as it is linked with mortality data in the National Civil Registration [27, 28]. In 2017, the University of the Witwatersrand in South Africa established its training institution alumni-based cohort, i.e. Wits longitudinal Study to Determine the Operation of the labour Market among its health professional graduates (WiSDOM) [21]. This includes eight cadres of health professionals and they have completed at least three rounds of follow-up with an around 80% response rate. Researchers of the WiSDOM study also documented the significant time and resources needed to set up the cohort study, including 280 h of consultations with professional councils, training institution class representatives, and academic heads of departments [21].

One additional contribution of this review is that we also summarised the major areas assessed and tracked as well as common measurement tools used in these longitudinal studies (see Table 2 for tools used three times or more). Most of the included studies examined workforce issues, however there was a lack of widely used measurement tools. It is understandable that questions on current job locations or intention-to-leave are context-specific and should be designed ad-hoc. However, issues related to work and job environment could be more accurately measured using standardised instruments with testing of psychometric properties. Out of the four commonly used workforce measurement tools, two of them (Copenhagen psychosocial questionnaire [COPSOQ] and Utrecht Work Engagement Scale) have been translated into over 20 languages and are free for academic use. In comparison, more standardised tools have been used to measure healthcare professionals’ wellbeing. Most of these tools are free and have been used in the general population in different contexts. We strongly recommend using and continued testing of tools in Table 2 to better capture experiences related to workforce and wellbeing and allowing comparisons, when designing surveys for longitudinal tracking.

While this study provides a comprehensive review of existing efforts to longitudinally track health professionals, several limitations should be considered while interpreting these findings. To start with, we only included studies that focused on doctors, nurses, midwives, and physician associates/assistants, and we recognise others that have tracked pharmacists and other allied health professionals [47, 48] which would be of relevance to broad workforce planning. Second, due to time and resource limitations we were only able to include articles published in English. Last but not least, our review is limited to studies reported in the research literature, and we acknowledge other tracking initiatives conducted by governments and regulators that did not lead to research publications [7]. For example Most US states operate a license-based survey system that requires doctors to report their practice location and characteristics upon their license renewal [49]. Similarly we were only able to extract data from the research articles and where possible protocols and reports available as appendices or on public websites, therefore information on follow-up and data linkage details, and measurement tools or questions used for surveys were missing for several studies.

This review has important implications for researchers, medical regulators and educators, and policy makers. When longitudinally tracking longer-term workforce outcomes of healthcare professionals, we recommend a mix of cohort and data linkage approaches, as leveraging existing secondary data improves cost-efficiency and reduces the burden on participants, but there is still value in conducting separate surveys as they could be used to understand and address emerging and specific workforce challenges, especially answering the *how* and *why* questions. Other approaches, such as baseline and short repeated measures, could be useful for specific research questions, particularly when assessing mental or physical health over shorter periods. We recommend collecting data across the different stages of the ‘working lifespan’, from workforce entry to exit. We also recommend using and continued testing of standardised measurement instruments to better capture experiences related to workforce and wellbeing and enable cross-site comparisons. In Table 1 we also present selected examples of longitudinal tracking studies in different countries that focused on different cadres of healthcare professionals and were differed in their recruitment and follow-up approaches. While this is only a shortlist of examples and the full list of all studies is provided in additional file 2, we hope researchers, medical educators, human resource managers and policy makers can learn from different options and consider the most appropriate surveying and tracking mechanisms in their settings. Additionally, understanding how these tracking studies have effectively contributed to workforce planning and policy changes is crucial and represents an important area for future research.

## Conclusion

In conclusion, we identified and described a large number of different healthcare professional longitudinal tracking studies. We recommend a mix of cohort and data linkage methods, collected across the workforce ‘working lifespan’, to improve cost-efficiency while capturing essential workforce and wellbeing experiences. Standardised measurement instruments should be further tested and utilised for better cross-site comparisons and insights into emerging workforce challenges.

## Supplementary Information

Additional file 1: Microsoft word document (.doc); Search strategy.

Additional file 2: Microsoft word document (.doc); Full list of studies.

Additional file 3: Microsoft word document (.doc); Detail on cohort and multiple cohort studies.

## Data Availability

All data relevant to the study are included in the article or uploaded as additional files.
