# Supplementary material for: Longitudinal tracking of healthcare professionals: a methodological scoping review
Source: BMC Med Res Methodol. 2025 Apr 1;25:83. doi: 10.1186/s12874-025-02533-1 (PMC11959867; doi:10.1186/s12874-025-02533-1)
Supplement: Supplementary file 1 — Additional file 1: Microsoft word document (.doc); Search strategy. [file 12874_2025_2533_MOESM1_ESM.docx]

**Additional file 1. Search strategy**

Database(s): Embase 1974 to present
Search Strategy:

| **#** | **Searches** | **Results** |
| --- | --- | --- |
| 1 | exp cohort analysis/ | 1001128 |
| 2 | cohort*.tw. | 1432891 |
| 3 | 1 or 2 | 1626459 |
| 4 | exp medical personnel/ | 1193541 |
| 5 | (nurse* or nursing or physician* or doctor* or "medical officer*" or clinician* or "clinical officer*" or specialist* or midwi*).ti,ab. | 1839642 |
| 6 | 4 or 5 | 2553351 |
| 7 | career/ | 32196 |
| 8 | exp workforce/ | 14620 |
| 9 | (career or employment or labour or labor or job or workforce or "human resource*" or migrat* or emigrat* or salar* or occupation or pay or absenteeism).ti,ab. | 997549 |
| 10 | 7 or 8 or 9 | 1016276 |
| 11 | 3 and 6 and 10 | 8509 |
| 12 | limit 11 to (english language and yr="2000 - 2022") | 7984 |
