# Supplementary material for: Longitudinal tracking of healthcare professionals: a methodological scoping review
Source: BMC Med Res Methodol. 2025 Apr 1;25:83. doi: 10.1186/s12874-025-02533-1 (PMC11959867; doi:10.1186/s12874-025-02533-1)
Supplement: Supplementary file 2 — Additional file 2: Microsoft word document (.doc); Full list of studies. [file 12874_2025_2533_MOESM2_ESM.docx]

**Additional file 2. Full list of all studies**

| Category | Name of the cohort | Might be still ongoing | Country or territory | Study population | Rounds of "cohort" | Rounds of follow-up for the first cohort (excluding baseline) | Baseline year of the first cohort | Baseline recruitment | Baseline representativeness description | Baseline platform | Baseline size | Follow-up platform | Follow-up retention strategy / motivation | Most recent availabla follow-up size for the first cohort | Most recent availabla follow-up year for the first cohort | Data linkage to | Data linkage via | Workforce | Tools used | Wellbeing | Tools used | Physical health | Tools used |
| --- | --- | --- | --- | --- | --- | --- | --- | --- | --- | --- | --- | --- | --- | --- | --- | --- | --- | --- | --- | --- | --- | --- | --- |
| Cohort + followup | Chinese Nurses' Early eXit sTudy |  | China | Nurse | 1 | 1 | 2007 | Health facility | 12 institutions, not nationally representative | N/A | 3088 | N/A | N/A | 2854 | 2008 | N/A | Unique ID (initials of mother/father's names and DoB) | X | Copenhagen psychosocial questionnaire | X | Copenhagen burnout inventory | X | SF-8 |
|  | Danish Nurse Cohort | X | Denmark | Nurse | 1 | 2 | 1993 | Trade union | Census, nationally representative | Mail | 19898 | Mail | N/A | 12955 | 2009 | All national registry | National ID | X |  | X | Cohen's stress scale | X |  |
|  | Early Career Nurse Employment Experience Survey (ECNEES) | X | Australia | Nurse | 1 | 2 | 2016 | Training institutions | Two institutions, not nationally representative | Online link | 293 | Email and SMS | Financial, social media, certificate | 194 | 2017 | N/A | Unique ID (masking contact information) | X | Casey-Fink readiness for practice, | X | Casey-Fink nurse rentention survey |  |  |
|  | Finnish Health Care Professionals Study | X | Finland | Doctor | 1 | 2 | 2006 | Professional association | Randomly sampled, nationaly representative | Mail | 2841 | Email and mail | Email and postal reminder | 1462 | 2015 | N/A | N/A | X |  | X |  |  |  |
|  | Korea Nurses' Health Study | X | South Korea | Nurse | 1 | 10 | 2013 | Health facility | Convenience sample, not nationally representative | Online link | 20613 | SMS | Financial | 10253 | 2020 | N/A | Registration ID and name | X |  | X | PHQ-9, PSS, Chalder Fatigue Scale, Spielberger State-Trait Anxiety Inventory | X | Jenkins Sleep Questionnaire |
|  | Medical Student Cognitive Habits and Growth Evaluation (CHANGE) | X | US | Doctor | 1 | 3 | 2010 | Training institutions | 49 institutions, not nationally representative | Paper and online link | 4732 | N/A | Financial | 3292 | 2016 | N/A | N/A | X |  | X | MBI, PROMIX Anxiety Scale, PSS | X |  |
|  | MedTrack | X | Ireland | Doctor | 1 | 1 | 2016 | Training institutions | Open to all, nationally representative | Online link | 483 | Email | N/A | N/A | N/A | N/A | N/A | X |  | X |  |  |  |
|  | MEMO (Minimizing Error, Maximizing Outcome) Study |  | US | Doctor | 1 | 1 | 2002 | Health facility | Three states, not nationally representative | Email or phone | 422 | N/A | N/A | N/A | N/A | N/A | N/A | X | Global job satisfaction measure, Kralweski's organisational climate, Trust in the organization scale, Workplace cohesiveness scale, Values alignment between physicians and leadership scale | X | Job stress measure |  |  |
|  | Munich Physician Cohort |  | Germany | Doctor | 1 | 3 | 2003 | Professional registry | Subnational sample, not national representative | Mail | 621 | Mail | Mail reminder | 373 | 2014 | N/A | N/A | X | Utrecht Work Engagement Scale, TAA-KH-S (job resource and demand) | X | Spielberger's state-trait depression scale. MBI |  |  |
|  | Newly Educated Workers |  | Finland | Nurse | 1 | 2 | 2013 | Professional registry | Open to all, nationally representative | Email | 318 | Email | N/A | 93 | 2015 | N/A | N/A | X | practice environment scale - Nursing Work Index (PES-NWI), Hospital Ethical Climate Survey, Occupational Commitment Scale, Qualities of Empowered Nurse Scale |  |  |  |  |
|  | Survey of Shift work, Sleep and Health (SUSSH) | X | Norway | Nurse | 1 | 11 | 2008 | Professional association | Convenience sample, not nationally representative | Mail | 2059 | Email and mail | Mail reminder, prize lottery | 1532 | 2020 | N/A | Unique ID | X | Negative Acts Questionnaire, Job demand control scale, Job Satisfaction Index | X | Hospital Anxiety and Depression Scale, Fatigue Questionnaire, Dispositional Resilience Scale-15. | X | SF-12, |
|  | NOvice Nurses 2-year follow-UP (NON2UP) |  | South Korea | Nurse | 1 | 5 | 2014 | Health facility | Single institution, not nationally representative | Paper | 492 | Paper | Gift | 355 | 2015 | N/A | N/A | X |  | X | Korean Center for Epidemiologic Studies-Depres- sion scale | X | General Sleep Disturbance Scale |
|  | Nurses and Midwives e-Cohort Study (NMeS) | X | Australia, New Zealand and UK | Nurse, midwife | 1 | 3 | 2006 | Professional registry | Statistically representative of the general nursing workforce | Mail | 10721 | Email | Financial, email reminder | 4991 | 2010 | N/A | Unique ID | X | Job content scale, Effort-Reward Imbalance subscale, Hostality socre | X | CES-D | X | IPAQ, SF-36, Diet Quality Score |
|  | Nurses Worklife and Health Study (NWHS) Part 3 | X | US | Nurse | 1 | 2 | 2002 | Professional registry | Randomly sampled, nationaly representative | Mail | 2624 | Mail | Financial | 2257 | 2004 | N/A | N/A | X | Standard Shiftwork Index, Job Content Questionnaire |  |  |  |  |
|  | Nurses' Early Exit Study (NEXT) |  | 10 countries | Nurse | 1 | 1 | 2002 | Health facility | Randomly sampled, nationaly representative | Paper | 77681 | N/A | N/A | 57819 | 2003 | N/A | Unique ID | X | Copenhagen Psychosocial Questionnaire, Work Ability Index |  |  | X | SF-6 |
|  | ORganisation des SOins - SAnté des soignants (ORSOSA) |  | France | Nurse, nurse assistant | 1 | 1 | 2006 | Health facility | 7 subnational units, not nationally representative | N/A | 4350 | N/A | N/A | 3424 | 2008 | N/A | N/A | X | Nursing Work Index (NWI-EO), Siegrist's effort-reard imbalance | X | Center for Epidemiologic Studies-Depression, State-trait anxiety inventory, nursing stress index | X |  |
|  | Promoting Physical and Mental Health of Caregivers II (Pro-Care II) |  | US | Nurse assistant | 1 | 1 | 2012 | Care facility | 24 institutions, not nationally representative | N/A | 2642 | N/A | Financial | 1237 | 2013 | N/A | N/A | X | Job Content Questionnaire | X | Center for Epidemiological Studies Depression |  |  |
|  | SwissMedCareer Study |  | Switzerland | Doctor | 1 | 5 | 2001 | Training institutions | Three institutions, not nationally representative | Mail | 711 | Mail | N/A | 526 | 2010 | N/A | Unique ID | X | Effort–Reward Imbalance at Work Questionnaire, Mentor-Prote´ge´ Relationships Questionnaire | X | Trier Inventory for the Assessment of Chronic Stress |  |  |
|  | The Nightingale study | X | Netherlands | Nurse | 1 | 2 | 2011 | Professional registry | Open to all, nationally representative | Mail | 59947 | N/A | Gift | 37731 | 2017 | N/A | Unique ID | X |  |  |  | X |  |
|  | Training for Health Equity Network Graduate Outcome Study (GOS) |  | 6 countries | Doctor | 1 | 1 | 2012-2016 | Training institutions | Eight institutions, not nationally representative | Paper and online link | 2557 | Paper and online link | N/A | N/A | N/A | N/A | N/A | X |  |  |  |  |  |
|  | Well-being in Hospital Employees Cohort (WHALE) | X | Denmark | HCW | 1 | 1 | 2011 | Health facility | One subnational, not nationally representative | Paper and email | 28759 | Email and paper | N/A | 19572 | 2014 | Employer based administrative registers and national registers | Unique ID | X | Copenhagen Psychosocial Questionnaire II | X |  |  |  |
|  | WiSDOM (Wits longitudinal Study to Determine the Operation of the labour Market among its health professional graduates) | X | South Africa | HCW | 1 | 3 | 2017 | Training institutions | Single institution, not nationally representative | Online link | 457 | Email and SMS | Financial, reminder | 333 | 2020 | N/A | N/A | X |  |  |  |  |  |
|  | Working Minds |  | UK | Nurse | 1 | 3 | 1994 | Training institutions | Not nationally representative | N/A | 359 | N/A | N/A | 192 | 1997 | N/A | N/A |  |  | X | GHQ-28, brief life events inventory, work-stress inventory |  |  |
|  | Graduate e-Cohort (GeS) | X | Australia, New Zealand, Canada and Ireland | Nurse, midwife | 1 | 4 | 2008 | Training institutions | Three institutions, not nationally representative | Online link | 111 | N/A | N/A | N/A | N/A | N/A | N/A | X |  |  |  |  |  |
|  | McManus C et al. Westminster medical school cohort |  | UK | Doctor | 1 | 1 | 1988 | Training institutions | Single institution, not nationally representative | N/A | 464 | Mail | N/A | 348 | 2001 | N/A | N/A | X |  | X | aMBI, GHQ-12 |  |  |
|  | Galvin J et al. UK nursing cohort |  | UK | Nurse | 1 | 1 | 2014 | Training institutions | Single facility, not nationally representative | Paper | 573 | Paper | N/A | 347 | 2015 | N/A | N/A |  |  | X | Wellbeing Process Questionnaire, Nurse Stress Scale |  |  |
|  | Sundin L et al. Sweden nursing student cohort |  | Sweden | Nurse | 1 | 1 | N/A | Health facility | Three institutions, not nationally representative | Mail | 2330 | Mail | Mail reminder | N/A | N/A | N/A | N/A | X |  | X | MBI |  |  |
|  | Schindler A Germany medical student cohort |  | Germany | Doctor | 1 | 3 | 2019 | Training institutions | Single facility, not nationally representative | Paper | 84 | Paper and online link | N/A | 63 | 2020 | N/A | N/A | X | DREEM | X | PHQ-9, FAHW-12, MBI |  |  |
|  | Gelsema T et al. Netherlands nurse cohort |  | Netherlands | Nurse | 1 | 1 | N/A | Health facility | Single facility, not nationally representative | N/A | 807 | N/A | N/A | N/A | N/A | N/A | N/A | X | Leiden Quality of Work Life Questionnaire for Nurse | X | MBI, scl-90 |  |  |
|  | Halpin Y et al. UK nursing student cohort |  | UK | Nurse | 1 | 2 | 2010 | Training institutions | Single institution, not nationally representative | Online link | 288 | Online link | N/A | 86 | 2011 | N/A | N/A | X |  | X | Nursing Stress Scale |  |  |
|  | RN Work Project |  | US | Nurse | 1 | 5 | 2006 | Professional registry | Subnational sample, not national representative | Mail | 3266 | Mail | Financial | 1080 | 2015 | N/A | N/A | X |  | X |  |  |  |
|  | Watson R et al. Hongkong nursing student cohort |  | Hongkong | Nurse | 1 | 1 | 2004 | Training institutions | Single institution, not nationally representative | Paper | 158 | Paper | N/A | 147 | 2005 | N/A | N/A |  |  | X | GHQ-12, MBI, Coping inventory for stressful situations, stress in nursing students |  |  |
|  | Sarfraz M et al. UK doctor cohort |  | UK | Doctor | 1 | 1 | N/A | Training institutions | Subnational sample, not national representative | Paper | 101 | Online link | N/A | N/A | N/A | N/A | N/A | X |  |  |  |  |  |
|  | Walkiewicz M et al. medical student cohort |  | Poland | Doctor | 1 | 6 | 1999 | Training institutions | Single institution, not nationally representative | N/A | 178 | N/A | N/A | 138 | 2005 | N/A | N/A | X |  | X | MMPI-D, Spielberger's State-Trait Anxiety inventory, MBI |  |  |
|  | Hatch D et al. US nurse cohort |  | US | Nurse | 1 | 1 | 2015 | Health facility | Single institution, not nationally representative | Email | 402 | Email | N/A | 352 | 2016 | N/A | N/A | X | Work Ability Index | X | OLBI | X |  |
|  | Tanaka M et al. Japan nurse cohort |  | Japan | Nurse | 1 | 1 | N/A | Health facility | Single institution, not nationally representative | Paper | 831 | Paper | N/A | N/A | N/A | N/A | Unique ID | X |  | X | Nursing Stress Scale, HADS |  |  |
|  | Liebermann S et al. Germany nurse cohort |  | Germany | Nurse | 1 | 1 | 2010 | Health facility | Single institution, not nationally representative | N/A | 387 | N/A | N/A | 345 | 2011 | N/A | Unique ID (five digit code) | X |  |  |  |  | SF-36 |
|  | Bratt M et al. US nurse cohort |  | US | Nurse | 1 | 2 | 2005 | Health facility | Subnational sample, not national representative | N/A | N/A | N/A | N/A | N/A | N/A | N/A | N/A | X | Nurse job satisfaction scale | X | Job stress scale |  |  |
|  | Selamu M et al. Ethippia HCW cohort |  | Ethiopia | HCW | 1 | 1 | 2014 | Health facility | Subnational sample, not national representative | Paper | 145 | Paper | N/A | 145 | 2014 | N/A | N/A | X | Job satisfaction questionnaire, Oslo social support scale, job content questionnaire | X | MBI, PHQ-9 | X |  |
|  | de Looff P et al. Netherlands nurse cohort |  | Netherlands | Nurse | 1 | 3 | 2015 | Health facility | Not nationally representative | N/A | 110 | N/A | N/A | 68 | 2017 | N/A | N/A | X | Demands and Support questionnaire | X | MBI |  |  |
|  | Bakker D et al. Canada nurse cohort |  | Canada | Nurse | 1 | 1 | 2004 | Health facility | All oncology nurse, nationally representative | N/A | 615 | N/A | N/A | 397 | 2006 | N/A | N/A | X | Revised Nursing Work Index |  |  |  |  |
|  | Miers M et al. UK HCW cohort |  | UK | HCW | 1 | 1 | 2001 | Training institutions | Single institution, not nationally representative | Paper | 821 | Paper | N/A | N/A | N/A | N/A | Unique ID | X |  |  |  |  |  |
|  | Shortland D et al. UK paediatrician cohort |  | UK | Doctor | 1 | 1 | 2009 | Training institutions | Single institution, not nationally representative | Email | 352 | Email | N/A | 308 | 2011 | N/A | N/A | X |  |  |  |  |  |
|  | Robinson et al. UK nurse cohort |  | UK | Nurse | 1 | 3 | 1998 | Training institutions | All nurses, nationally representative | Paper and online link | 3476 | Mail | Decision on interval, reminder, postage stamps | 1785 | 2001 | N/A | N/A | X |  |  |  |  |  |
|  | Cowin L et al. Australia nurse cohort |  | Australia | Nurse | 1 | 1 | 2002 | Professional registry | Subnational sample, not national representative | Mail | 528 | Mail | N/A | 332 | 2003 | N/A | N/A | X | index of work satisfaction, nurse retention index, nurses' self-concept questionnaire |  |  |  |  |
|  | Adriaenssens J et al. Netherlands nurse cohort |  | Netherlands | Nurse | 1 | 1 | 2008 | Health facility | Randomly sampled, nationaly representative | Paper | 254 | Paper | N/A | 170 | 2009 | N/A | Unique ID | X | Leiden Quality of Work Questionnaire, Utrecht Work Engagement Scale | X | MBI, Brief Symptom Inventory |  |  |
|  | Maidment R et al. UK doctor cohort |  | UK | Doctor | 1 | 1 | N/A | Training institutions | Single institution, not nationally representative | Mail | 379 | Mail | Reminder | N/A | N/A | N/A | N/A | X |  |  |  |  |  |
|  | Rognstad M and Aasland O Norway nurse cohort  and Aasland, Olaf |  | Norway | Nurse | 1 | 2 | 1998 | Training institutions | Single institution, not nationally representative | Paper | 315 | Mail | Phone interview as supplement | 140 | 2003 | N/A | N/A | X |  |  |  |  |  |
|  | Nielsen B et al. Denmark nurse cohort |  | Denmark | Nurse | 1 | 1 | 2020 | Social media | Uncertainty about representativeness | Online link | 1165 | Online link | N/A | 426 | 2020 | N/A | N/A | X |  | X | PHQ-9, GAD-7, PSS-10 | X | Insomnia severity index |
|  | Institute for Studies of the Medical Profession in Norway (LEFO) doctor cohort | X | Norway | Doctor | 1 | 11 | 1994 | Professional association | All doctors, national representation | Mail | 1272 | Mail | N/A | 640* | 2016 | N/A | N/A | X | Warr, Cook and Wall Job satisfaction scale | X | ERI | X |  |
|  | Dyrbye L et al. US HCW cohort |  | US | HCW | 1 | 1 | 2015 | Health facility | Subnational sample, not national representative | Email | 26292 | Email | N/A | 26280 | 2017 | N/A | N/A | X |  | X | MBI |  |  |
|  | Chen D et al. US medical student cohort |  | US | Doctor | 1 | 4 | 2007 | Training institutions | Single institution, not nationally representative | Email | N/A | Email | N/A | N/A | N/A | N/A | N/A | X |  |  |  |  |  |
|  | Boonluksiri P et al. Thailand doctor cohort |  | Thailand | Doctor | 1 | 1 | 2010 | N/A | N/A | N/A | N/A | N/A | N/A | N/A | N/A | N/A | N/A | X |  |  |  |  |  |
|  | Yanos P et al. US HCW cohort |  | US | HCW | 1 | 1 | N/A | Health facility | Single facility, not nationally representative | Email | 68 | Email | Financial | N/A | N/A | N/A | N/A | X |  | X | MBI |  |  |
|  | Mantler J et al. Canada nurse cohort |  | Canada | Nurse | 1 | 2 | 2001 | Professional association | Subnational sample, not national representative | Mail | 833 | Mail | Reminder | N/A | N/A | N/A | Unique ID (participant self-generated) | X |  |  |  |  |  |
|  | Davis B et al. US medical student cohort |  | US | Doctor | 1 | 2 | 1993 | Training institutions | Three institutions, not nationally re representative | N/A | 695 | N/A | N/A | 502 | 1997 | N/A | Social security number | X |  |  |  |  |  |
|  | Sullivan A et al. US doctor cohort |  | US | Doctor | 1 | 1 | 2020 | Health facility | Single facility, not nationally representative | Email | 182 | Email | N/A | 149 | 2021 | N/A | N/A | X |  | X | Mini-Z |  |  |
|  | Davison I et al. UK doctor cohort |  | UK | Doctor | 1 | 1 | 2001 | Training institutions | Three institutions, not nationally representative | N/A | 72 | Mail | N/A | 40 | 2003 | N/A | N/A | X |  |  |  |  |  |
|  | Frank E et al. medical student cohort |  | US | Doctor | 1 | 2 | 2003 | Training institutions | Intended to representative | Mail | 2316 | Mail | Dillman’s five stage mailing process | 1393 | 2006 | N/A | Unqie ID (masking mother's initial at birth and father's first two initials) | X |  |  |  |  |  |
|  | Ayas N et al. US doctor cohort |  | US | Doctor | 1 | N/A | 2002 | Training institutions | Uncertainty about representativeness | Email | 2737 | Email | Reminder | N/A | N/A | N/A | N/A | X |  |  |  | X |  |
|  | Baldwin K et al. US doctor cohort |  | US | Doctor | 1 | 5 | 2005 | Training institutions | Single institution, not nationally representative | Email | 345 | Email | N/A | N/A | N/A | N/A | N/A | X |  |  |  |  |  |
|  | Liu S et al. China doctor cohort |  | China | Doctor | 1 | 1 | 2013 | Health facility | Subnational sample, not national representative | Paper | 153 | Paper | N/A | 142 | 2016 | N/A | N/A | X |  |  |  |  |  |
|  | Playford D et al. Australia HCW study |  | Australia | HCW | 1 | 1 | 2000 | Training institutions | Subnational sample, not national representative | Online link | 776 | Email and phone | N/A | 474 | 2001 | Regulator database | Name | X |  |  |  |  |  |
|  | Bedard M et al. Canada doctor cohort |  | Canada | Doctor | 1 | 1 | 2003 | Training institutions | Subnational sample, not national representative | N/A | 485 | N/A | N/A | 353 | 2005 | N/A | N/A | X |  |  |  |  |  |
|  | Guilfoyle C et al. Australia doctor cohort |  | Australia | Doctor | 1 | N/A | 2012 | Training institutions | Single institution, not nationally representative | N/A | N/A | N/A | N/A | N/A | N/A | N/A | N/A | X |  |  |  |  |  |
|  | Sigurdardottir A et al. Iceland nurse cohort |  | Israel | Nurse | 1 | 2 | 2009 | Health facility | Single institution, not nationally representative | Paper and email | 162 | Email and paper | N/A | 156 | 2011 | N/A | N/A | X | Demand–Control Support Questionnaire |  |  |  |  |
|  | Cowin L and Johnson M Australia nurse cohort |  | Australia | Nurse | 1 | 3 | 2009 | Training institutions | Single institution, not nationally representative | N/A | 676 | Online link | N/A | 190 | 2012 | N/A | N/A | X |  |  |  |  |  |
|  | Alers M et al. Netherlans medical student cohort |  | Netherlands | Doctor | 1 | 1 | 2006 | Training institutions | Single institution, not nationally representative | Paper | N/A | Online link | N/A | 292 | 2009 | N/A | N/A | X |  |  |  |  |  |
|  | May J et al. Australia GP cohort |  | Australia | Doctor | 1 | 1 | 2005 | Government authority | Subnational sample, not national representative | Face-to-face interview | 31 | Face-to-face interview | Face-to-face interview | 29 | 2006 | N/A | N/A | X |  |  |  |  |  |
|  | Takase J et al. Japan nurse cohort |  | Japan | Nurse | 1 | 3 | N/A | Health facility | Five facilities, not nationally representative | Mail | 150 | Mail | N/A | N/A | N/A | N/A | N/A | X |  |  |  |  |  |
|  | Cohen M et al. Canada doctor cohort |  | Canada | Doctor | 1 | 1 | 1993 | Professional registry | Subnational sample, not national representative | Mail | 550 | Mail | N/A | 293 | 1999 | N/A | N/A | X |  |  |  |  |  |
|  | Ahmed F et al. China nurse cohort |  | China | Nurse | 1 | 2 | 2020 | Health facility | Five facilities, not nationally representative | Online link | 497 | Online link | N/A | 451 | 2020 | N/A | N/A | X | Edmondson psychological safert, Carmeli iclusive leadershio | X | K6 |  |  |
|  | Zhang Y et al. China nurse cohort |  | China | Nurse | 1 | 3 | 2014 | Health facility | Four facilities, not nationally representative | Paper | 332 | Paper | N/A | 203 | 2017 | N/A | N/A | X |  | X | Yeh and Yu occupational stress scale |  |  |
|  | Van der Heijden B et al. Netherlands nurse cohort |  | Netherlands | Nurse | 1 | 1 | N/A | Health facility | Subnational sample, not national representative | Paper or mail | N/A | Paper or mail | N/A | N/A | N/A | N/A | N/A | X | COPSOQ | X | CBI |  |  |
|  | Hogh A et al. Denmark HCW cohort |  | Denmark | HCW | 1 | 1 | 2006 | Health facility | All eldercare, national representative | Paper | 9212 | N/A | N/A | 5204 | 2008 | N/A | N/A | X | COPSOQ | X |  |  |  |
|  | Matsuo M et al. Japan nurse cohort |  | Japan | Nurse | 1 | 1 | 2017 | Health facility | Selected facilities, not nationally representative | Paper | 1368 | Paper | N/A | 975 | 2018 | N/A | N/A | X |  | X | MBI |  |  |
|  | Kim E and Kim S South Korea nurse cohort |  | South Korea | Nurse | 1 | 2 | 2016 | Training institutions | Five facilities, not nationally representative | Paper | 526 | Online link | N/A | 338 | 2020 | N/A | N/A | X |  |  |  |  |  |
|  | Riese H et al. Netherland nurse study |  | Netherlands | Nurse | 1 | 1 | N/A | Health facility | Three facilities, not nationally representative | Paper | 662 | Paper | N/A | N/A | N/A | N/A | N/A | X |  | X | STAI, CES-D | X |  |
|  | Cheng C et al. Taiwan nurse cohort |  | Taiwan | Nurse | 1 | 2 | N/A | Training institutions | Single institution, not nationally representative | Paper or mail | 117 | Paper or mail | N/A | N/A | N/A | N/A | N/A | X | Job/work environment nursing satisfaction survey (WENSS) | X | Clinical stress scale |  |  |
|  | Zhao X et al. China HCW cohort |  | China | HCW | 1 | 1 | 2020 | Health facility | Single facility, not nationally representative | N/A | 230 | N/A | N/A | 215 | 2020 | N/A | N/A | X |  | X |  | X |  |
|  | Blazey-Martin D et al. US doctor cohort |  | US | Doctor | 1 | N/A | 1995 | Training institutions | Randomly sampled, nationaly representative | N/A | 1790 | Email | N/A | 607 | 2012 | Web-based search | Name | X |  |  |  |  |  |
|  | Gillet N et al. France nurse cohort |  | France | Nurse | 1 | 1 | 2015 | Health facility | Eight institutions, not nationally representative | N/A | 410 | Email | N/A | 294 | 2016 | N/A | N/A | X | Utrecht Work Engagement Scale |  |  |  |  |
|  | Munir F et al. Denmark HCW cohort |  | Denmark | HCW | 1 | 1 | N/A | Health facility | Single facility, not nationally representative | N/A | 447 | N/A | N/A | N/A | N/A | N/A | N/A | X | COPSOQ | X |  |  |  |
|  | Ladha F et al. US medical student cohort |  | US | Doctor | 1 | 3 | 2017 | Training institutions | Single institution, not nationally representative | Paper | 76 | Email and paper | N/A | 64 | 2020 | N/A | N/A | X |  |  |  |  |  |
|  | Zhou P et al. China HCW cohort |  | China | HCW | 1 | 1 | 2020 | Health facility | Subnational sample, not national representative | Online link | 1260 | Online link | N/A | 963 | 2021 | N/A | N/A | X |  | X | PHQ-9, GAD | X |  |
|  | Topa G et al. Spain nurse cohort |  | Spain | Nurse | 1 | 1 | 2010 | Health facility | Two institutions, not nationally representative | Paper | 361 | Paper | N/A | 201 | 2010 | N/A | Unique ID | X | Negative Mentoring Experiences | X | Negative Acts Questionnaire |  |  |
|  | Tanner G et al. Germany doctor cohort |  | Germany | Doctor | 1 | 1 | N/A | Health facility | 14 institutions, not nationally representative | Paper and email | 298 | Email and paper | N/A | N/A | N/A | N/A | Unique ID | X | Instrument for Stress‐Related Job Analysis for Hospital Physicians | X |  |  |  |
|  | Gasiorowsk J et al. Poland medical student cohort |  | Poland | Doctor | 1 | 1 | 2003 | Training institutions | Single institution, not nationally representative | N/A | 143 | N/A | N/A | N/A | N/A | N/A | N/A | X |  |  |  |  |  |
|  | Miranda H et al. US HCW cohort |  | US | HCW | 1 | 2 | 2006 | Health facility | 12 institutions, not nationally representative | Paper or mail | 867 | Paper or mail | N/A | 344 | 2008 | N/A | N/A | X | Job Content Questionnaire |  |  | X |  |
|  | West S et al. Australia nurse cohort |  | Australia | Nurse | 1 | 2 | N/A | Training institutions | Single institution, not nationally representative | N/A | 150 | N/A | N/A | N/A | N/A | N/A | N/A | X | WOCR, job satisfaction scale | X | GHQ-28, MBI | x |  |
|  | Guerrero S et al. Canada nurse cohort |  | Canada | Nurse | 1 | 2 | N/A | Training institutions | Subnational sample, not national representative | Mail | 675 | Mail | N/A | N/A | N/A | N/A | N/A | X |  |  |  |  |  |
|  | Gaudine A and Thorne L Canada nurse cohort |  | Canada | Nurse | 1 | 1 | N/A | Health facility | 4 institutions, not nationally representative | Paper | N/A | Paper | N/A | N/A | N/A | Hospital employers's record | Unique ID | X | Meyer and Allen’s organisational commitment scale | X | Patchen stress scale |  |  |
|  | Kloster T et al. Norway nursing student cohort |  | Norway | Nurse | 1 | 1 | 2001 | Training institutions | 5 institutions, not nationally representative | N/A | 620 | N/A | N/A | 386 | 2003 | N/A | N/A | X |  |  |  |  |  |
|  | Ten Hoeve Y et al. Netherlands nursing student cohort |  | Netherlands | Nurse | 1 | 3 | 2011 | Training institutions | 4 institutions, not nationally representative | Paper | 1244 | Paper | N/A | 403 | 2013 | N/A | N/A | X | Nursing Orientation Tool, Nursing Attitude Questionnaire |  |  |  |  |
|  | Nesje K Norway nursing student cohort |  | Norway | Nurse | 1 | 2 | 2005 | Training institutions | Single institution, not nationally representative | Paper | N/A | Paper or mail | N/A | 84 | 2010 | N/A | N/A | X | Job involvement questionnaire |  |  |  |  |
|  | Tuomi J et al. Finland nursing student cohort |  | Finland | Nurse | 1 | 1 | 2010 | Training institutions | Single institution, not nationally representative | Paper | 258 | Paper | N/A | 135 | 2011 | N/A | Name | X |  |  |  |  |  |
|  | Meyer R et al. US nurse cohort |  | US | Nurse | 1 | 2 | 2007 | Health facility | Single institution, not nationally representative | Paper | 251 | Paper and online link | N/A | N/A | N/A | N/A | N/A | X | Mueller McCloskey Satisfaction Scale | X | Life Events Checklist, Compassion Fatigue Self Test |  |  |
|  | DeKeyser Ganz F and Kahana S Israel nursing student cohort |  | Israel | Nurse | 1 | 1 | 1998 | Training institutions | Single institution, not nationally representative | Paper | N/A | Paper | N/A | N/A | N/A | N/A | N/A | X |  |  |  |  |  |
|  | van Leeuwen E et al. Netherland doctor cohort |  | Netherlands | Doctor | 1 | 2 | 2019 | Health facility | 2 institutions, not nationally representative | Email | 165 | Email | N/A | 75 | 2020 | N/A | N/A | X | Work design questionnaire, VBBA 2.0 |  |  |  |  |
|  | Khan K et al. UK doctor cohort |  | UK | Doctor | 1 | 1 | 2007 | Training institutions | 2 institutions, not nationally representative | N/A | 46 | N/A | N/A | 45 | 2007 | N/A | N/A | X |  |  |  |  |  |
|  | Chande V and Krug S US doctor cohort |  | US | Doctor | 1 | 1 | 1994 | Professional association, registry | All paediatric emergency trainee, nationally representative | Mail | 183 | Mail | N/A | 170 | 1999 | N/A | N/A | X |  |  |  |  |  |
|  | Wagner C US nurse cohort |  | US | Nurse | 1 | 1 | 2005 | Health facility | 2 institutions, not nationally representative | N/A | 756 | N/A | N/A | 496 | 2006 | N/A | N/A | X | Organisational commitment questionnaire, job related tension index, anticipated turnover scale |  |  |  |  |
|  | Ayuso-Raya M et al. Spain doctor cohort |  | Spain | Doctor | 1 | 1 | 2005 | Training institutions | Single institution, not nationally representative | N/A | 79 | N/A | N/A | 76 | 2009 | Government website | N/A | X |  |  |  |  |  |
|  | Diehl A et al. US doctor cohort |  | US | Doctor | 1 | 1 | 1991 | Training institutions | 2 institutions, not nationally representative | N/A | 204 | Mail | N/A | 198 | 2002 | N/A | N/A | X |  |  |  |  |  |
|  | Fochsen G et al. Sweden nurse cohory |  | Sweden | Nurse | 1 | 1 | 1992 | Health facility | 2 institutions, not nationally representative | N/A | 1771 | Mail | Reminder | 1095 | 2003 | N/A | Civil registration number | X | Rating of Perceived Exertion scale |  |  | X |  |
|  | Garfinkel P et al. Canada psychiatry cohort |  | Canada | Doctor | 1 | 1 | 1974 | Training institutions | 2 institutions, not nationally representative | N/A | 70 | Mail | N/A | 29 | 1998 | N/A | N/A | X |  |  |  |  |  |
|  | Olive V et al. Spain doctor cohort |  | Spain | Doctor | 1 | 1 | 2017 | Health facility | Single facility, not nationally representative | Paper | 71 | Paper | N/A | 59 | 2018 | N/A | N/A |  |  | X | PSS, MBI | X |  |
|  | Yang L et al. US nurse cohort |  | US | Nurse | 1 | 1 | N/A | Health facility | 2 facilities, not nationally representative | Paper and online link | 471 | Paper and online link | Financial | N/A | N/A | N/A | N/A | X | Violence prevention climate scale |  |  | X |  |
|  | Amati M et al. Italy nurse cohort |  | Italy | Nurse | 1 | 3 | 2004 | Health facility | Single facility, not nationally representative | Face-to-face interview | N/A | Paper | N/A | N/A | N/A | N/A | N/A | X | Healthcare job satisfaction scale, Multidimensional Scale of Perceived Social Support | X | GHQ-12, PSS | X |  |
|  | Pathman D et al. US doctor cohort |  | US | Doctor | 1 | 1 | 1991 | Professional association | Sub-sample, not nationally representative | Mail | 699 | Mail and phone | N/A | 681 | 1996 | Professional association masterlist | N/A | X |  |  |  |  |  |
|  | Ares T US nurse cohort |  | US | Nurse | 1 | 1 | N/A | Professional associations | Not nationally representative | N/A | 113 | N/A | N/A | N/A | N/A | N/A | N/A | X |  |  |  |  |  |
|  | Kenny P et al. Australia nurse cohort |  | Australia | Nurse | 1 | N/A | 2008 | Training institutions | 2 institutions, not nationally representative | Online link | 503 | N/A | Gift | N/A | N/A | N/A | N/A | X |  |  |  | X |  |
|  | Terry D et al. Australia nurse cohort |  | Australia | Nurse | 1 | 1 | N/A | Training institutions | Single institution, not nationally representative | Email | 62 | Email | N/A | N/A | N/A | N/A | Birth date and postcode | X | GSE-10 |  |  |  |  |
|  | Maiorova T et al. Netherlands doctor cohort |  | Netherlands | Doctor | 1 | 1 | 2002 | Training institutions | Single institution, not nationally representative | Mail | 852 | N/A | N/A | N/A | N/A | N/A | N/A | X |  |  |  |  |  |
|  | Stromgren M et al. Sweden HCW cohort |  | Sweden | HCW | 1 | 1 | 2012 | Health facility | 5 facilities, not nationally representative | Email or mail | 865 | Email and mail | Reminder | 477 | 2013 | N/A | N/A | X | COPSOQ II, Swedish Scale for Work Engagement and Burnout | X |  |  |  |
|  | Hagstrom T and Kjellberg A Sweden nurse cohort |  | Sweden | Nurse | 1 | 2 | 1993 | Training institutions | 4 institutions, not nationally representative | N/A | 719 | N/A | N/A | 384 | 1997 | N/A | N/A | X |  |  |  |  |  |
|  | Chai X et al. China nursing student cohort |  | China | Nurse | 1 | 2 | 2014 | Health facility | 6 facilities, not nationally representative | N/A | 351 | N/A | N/A | 313 | 2015 | N/A | N/A | X |  |  |  |  |  |
|  | Hebditch M et al. UK nursing student cohort |  | UK | Nurse | 1 | 3 | 2014 | Training institutions | 2 institutions, not nationally representative | N/A | 433 | N/A | N/A | 122 | 2016 | N/A | N/A | X |  |  |  |  |  |
|  | Bolan C and Grainger P Canada nursing student cohort |  | Canada | Nurse | 1 | 3 | 2001 | Training institutions | Single institutions, not nationally representative | N/A | 213 | N/A | N/A | 140 | 2004 | N/A | N/A | X |  |  |  |  |  |
|  | Goldin S et al. US medical student cohort |  | US | Doctor | 1 | N/A | 2005 | Training institutions | Single institutions, not nationally representative | N/A | N/A | N/A | N/A | N/A | N/A | N/A | N/A | X |  |  |  |  |  |
|  | Gupta S et al. Australia doctor cohort |  | Australia | Doctor | 1 | 9 | 2002 | Training institutions | 2 institutions, not nationally representative | Email or phone | N/A | Email and phone | Phone interview as supplement | N/A | N/A | N/A | N/A | X |  |  |  |  |  |
|  | Burke R Canada nurse cohort |  | Canada | Nurse | 1 | 1 | 1996 | Professional association | Subnational sample, not national representative | Mail | 1363 | Mail | N/A | 925 | 1999 | N/A | N/A | X | Quinn and Shepard Job satisfaction scale | X | Gneeral burnout questionnaire (Maslach 1997) | X |  |
|  | Davey B and Robinson S UK nurse cohort |  | UK | Nurse | 1 | 5 | 1990 | Training institutions | Subnational sample, not national representative | Mail | 1015 | Mail | N/A | 620 | 1998 | N/A | N/A | X |  |  |  |  |  |
|  | Doiron D and Yoo H Australia nurse cohort |  | Australia | Nurse | 1 | 1 | 2008 | Training institutions | 2 institutions, not nationally representative | N/A | N/A | N/A | N/A | 241 | 2009 | N/A | N/A | X |  |  |  |  |  |
|  | Veitch C et al. Australia doctor cohort |  | Australia | Doctor | 1 | 1 | 2001 | Training institutions | Single institutions, not nationally representative | N/A | 57 | N/A | N/A | 50 | 2005 | N/A | N/A | X |  |  |  |  |  |
|  | Palese A et al. Romanian/Italy nurse cohort |  | Italy | Nurse | 1 | 1 | 2004 | Health facility | Single institutions, not nationally representative | Face-to-face interview | 17 | Face-to-face interview | N/A | 10 | 2006 | N/A | N/A | X |  |  |  |  |  |
|  | Tzeng D et al. Taiwan nurse cohort |  | Taiwan | Nurse | 1 | 1 | 2005 | Health facility | 2 institutions, not nationally representative | N/A | N/A | N/A | N/A | 418 | 2006 | N/A | N/A | X | Job Content Questionnaire | X | GHQ-12 | X | WHOQOL-BREF |
|  | Magnavita N Italy HCW cohort |  | Italy | Nurse | 1 | 1 | 2003 | Health facility | Single institutions, not nationally representative | N/A | N/A | N/A | N/A | N/A | N/A | N/A | N/A | X | Job Content Questionnaire, Effort-Reward Imbalance subscale | X | Goldberg scale |  |  |
|  | Wolfson R et al. US medical student cohort |  | US | Doctor | 1 | 2 | 2014 | Training institutions | Single institutions, not nationally representative | N/A | N/A | N/A | N/A | 125 | 2015 | N/A | N/A | X |  |  |  |  |  |
|  | Ripp J et al. US medical resident cohort |  | US | Doctor | 1 | 1 | 2011 | Training institutions | 3 institutions, not nationally representative | N/A | 181 | N/A | N/A | N/A | N/A | N/A | N/A | X |  | X | MBI |  |  |
|  | Lee E and De Gagne J South Korea nurse cohort |  | South Korea | Nurse | 1 | 1 | 2019 | Health facility | Single institutions, not nationally representative | Online link | 219 | Online link | Email and phone reminder | 148 | 2020 | N/A | N/A | X | Cho et al. work environment; Shin et al. resilence |  |  |  |  |
|  | Salamonson Y ey al. Australia nursing student cohort |  | Australia | Nurse | 1 | 1 | 2006 | Training institutions | Single institutions, not nationally representative | N/A | 566 | N/A | N/A | 182 | 2008 | N/A | N/A | X |  |  |  |  |  |
|  | Kogan J et al. US medical student cohort |  | US | Doctor | 1 | 2 | 2006 | Training institutions | Single institutions, not nationally representative | N/A | 65 | N/A | N/A | N/A | N/A | N/A | N/A | X |  |  |  |  |  |
|  | Inoue M et al. Japan nurse cohort |  | Japan | Nurse | 1 | 1 | 2019 | Health facility | 2 institutions, not nationally representative | Paper | 695 | Paper | N/A | 583 | 2019 | N/A | Unique ID | X |  |  |  |  |  |
|  | Schalk R Netherlands nurse cohort |  | Netherlands | Nurse | 1 | 2 | N/A | Health facility | 2 institutions, not nationally representative | N/A | N/A | N/A | N/A | N/A | N/A | Hospital employers's record | N/A | X | Organizational Commitment Questionnaire |  |  |  |  |
|  | Chu L Taiwan HCW cohort |  | Taiwan | HCW | 1 | 1 | N/A | Health facility | 2 institutions, not nationally representative | Paper | 316 | Paper | N/A | N/A | N/A | N/A | Unique ID | X | Job Content Questionnaire | X | PSS, occupational stress indicator (Lu) |  |  |
|  | Chen F et al. Taiwan nurse cohort |  | Taiwan | Nurse | 1 | 2 | 2008 | Health facility | Single institutions, not nationally representative | Paper | 222 | Paper | N/A | N/A | N/A | N/A | N/A | X | Safety climate scale (Zohar and Luria) |  |  |  |  |
|  | Serpa A et al. Brazil HCW cohort |  | Brazil | HCW | 1 | 1 | 2020 | Government authority and social media | All HCW, nationally representative | Email | 205591 | N/A | N/A | 58218 | 2020 | N/A | N/A |  |  | X | Brief Symptom Inventory |  |  |
|  | Rabinowitz H et al. US doctor cohort |  | US | Doctor | 1 | N/A | 1978 | Training institutions | Single institution, not nationally representative | N/A | N/A | Mail | N/A | 762 | 2007 | N/A | N/A | X |  |  |  |  |  |
|  | Chenevert D et al. Canada nurse cohort |  | Canada | Nurse | 1 | 2 | 2009 | Professional association | Subnational sample, not national representative | Mail | 675 | Mail | N/A | 160 | 2012 | N/A | N/A | X |  |  |  |  |  |
|  | Fornes-Vives J et al. Spain nurse cohort |  | Spain | Nurse | 1 | 2 | 2007 | Training institutions | 3 institutions, not nationally representative | Paper | 249 | Email and paper | N/A | 70 | 2013 | N/A | N/A |  |  | X | Psychological Harassment from the Workplace |  |  |
|  | Zhang C and Liu Y China doctor cohort |  | China | Doctor | 1 | 1 | 2015 | Health facility | Randomly sampled, nationaly representative | Online link | 17975 | Online link | N/A | 2498 | 2016 | N/A | N/A | X |  |  |  |  |  |
|  | Myers D et al. US nurse cohort |  | US | Nurse, nurse assistant | 1 | 1 | N/A | Health facility | Single institution, not nationally representative | N/A | N/A | N/A | N/A | N/A | N/A | Hospital employers's record | N/A | X |  |  |  |  |  |
|  | Woolf K et al. UK medical student cohort |  | UK | Doctor | 1 | 1 | 2007 | Training institutions | Single institution, not nationally representative | N/A | 227 | Email | Email reminder, and prize | 210 | 2009 | N/A | N/A | X |  |  |  |  |  |
|  | Virtanen P and Koivisto A Finland medical student cohort |  | Finland | Doctor | 1 | 3 | 1994 | Training institutions | 5 institutions, not nationally representative | N/A | 79 | Mail | N/A | 69 | 1998 | N/A | N/A | X |  | X | GHQ |  |  |
|  | Khamisa N et al. South Africa nurse cohort |  | South Africa | Nurse | 1 | 1 | 2013 | Health facility | 4 institutions, not nationally representative | Paper | N/A | Paper | N/A | 277 | 2014 | N/A | N/A | X |  | X |  |  |  |
|  | Jacobsen H et al. US HCW cohort |  | US | HCW | 1 | 1 | 2009 | Health facility | 2 institutions, not nationally representative | Email | 840 | Email | N/A | 99 | 2011 | N/A | N/A | X |  |  |  | X |  |
|  | Guille C et al. US medical intern cohort |  | US | Doctor | 1 | 1 | 2015 | Training institutions | Selected facilities, not nationally representative | Email | 3121 | Email | Reminder and finance | N/A | N/A | N/A | N/A | X |  | X | PHQ-9 |  |  |
|  | Jones Lorelei and Fisher T UK doctor cohort |  | UK | Doctor | 1 | 9 | 1995 | Training institutions | All institutions providing training, nationaly presentative | Mail | 544 | Mail | N/A | 484 | 2004 | N/A | N/A | X |  |  |  |  |  |
|  | Safe at Work study |  | US | Nurse | 1 | 1 | 2007 | Health facility | 4 institutions, not nationally representative | Paper and online link | 2166 | Paper and online link | N/A | 1239 | 2008 | N/A | N/A | X |  | X |  |  |  |
|  | Hogh A et al. Denmark HCW cohort 2 |  | Denmark | HCW | 1 | 1 | 2004 | Training institutions | All HCW, nationally representative | Paper | 5696 | Mail | N/A | 3708 | 2005 | N/A | N/A |  |  | X |  |  |  |
|  | Williamson et al. New Zealand doctor cohort |  | New Zealand | Doctor | 1 | 1 | 2000 | Training institutions | Single institution, not nationally representative | N/A | 293 | Mail | N/A | 147 | 2009 | N/A | N/A | X |  |  |  |  |  |
| Multiple cohort + followup | Intern Health Study | X | US | Doctor | 14 | 4 | 2007 | Health facility | 14 institutions, not nationally representative | Email | 740 | Email | Financial | N/A | N/A | N/A | N/A |  |  | X | PHQ-9, GAD-7 | X |  |
|  | Longitudinal Analysis of Nursing Education (LANE) | X | Sweden | Nurse | 3 | 4 | 2002 | Government authority | Open to all, nationally representative | Mail | 4316 | Email and mail | N/A | 2474 | 2017 | N/A | N/A | X | Nordic Questionnaire of Psychosocial factors at work | X | Major depression inventory, oldenburg burnout inventory, life satisfaction scale, Copenhagen Psychosocial Questionnaire | X |  |
|  | Longitudinal Study of Emergency Medicine Residents | X | US | Doctor | 5 | 2 | 1996 | Health facility | Census, nationally representative | N/A | 159 | N/A | N/A | 1998 | N/A | N/A | N/A | X |  | X |  | X |  |
|  | Medical Schools Outcomes Database and Longitudinal Tracking (MSOD) | X | Australia and New Zealand | Doctor | 18 | 4 | 2005 | Training institutions | Census, nationally representative | Paper and online link | 878 | Email | N/A | N/A | N/A | Training institution database | Unique ID (masking contact information) | X |  |  |  |  |  |
|  | Medicine in Australia: Balancing Employment and Life | X | Australia | Doctor | N/A | 10 | 2008 | Professional association | Open to all, nationally representative | Mail | 10498 | Mail | Financial | 2352*(9361) | 2018 | National prescribing database | Unique ID | X | Warr-Cook-Wall Job Satisfaction Scale | X | Kessler K-6 |  |  |
|  | Longitudinal Study of Norwegian Medical Students and Doctors (NORDOC) | X | Norway | Doctor | 2 | 4 | 1993 | Training institutions | All four medical schools, nationally representative | Mail | 631 | Mail | Gift | 330 | 2008 | N/A | N/A | X | Climate for Learning | X | Cooper's Job Stress Questionnaire, Oldenburg Burnout Inventory | X |  |
|  | Nurses' Health Study | X | US | Nurse | 3 | 21 | 1976 | Professional association | 11 subnational units, not nationally representative | Mail (online for NHS3) | 121700 | Mail (online for NHS3) | Mail reminder | N/A | N/A | N/A | Unique ID | X | Job content questionnaire | X | Mental Health Index, Center for Epidemiologic Studies-Depression, Geriatric Depression Scale, Brief Trauma Questionnaire, Crown-Crisp Index | X |  |
|  | Pediatrician Life and Career Experience Study (PLACES) | X | US | Doctor | 3 | 10 | 2012 | Professional association | Open to all, nationally representative | Email or mail | 901 | Email and mail | Financial | 840 | 2013 | N/A | N/A | X |  | X | MBI |  |  |
|  | Thai Nurse Cohort Study | X | Thailand | Nurse | 2 | 1 | 2009 | Professional registry | Census, nationally representative | Mail | 18756 | Email and mail | Mail reminder | 11282 | 2012 | National Health and Death Registry | N/A | X | Job Content Questionnaire | X |  | X | IPAQ |
|  | Tracking Health Professional Students and Graduates Project |  | Netherlands | Doctor | 2 | 1 | 2006 | Training institutions | Single institution, not nationally representative | N/A | 216 | N/A | N/A | N/A | N/A | Regulator database | Unique ID | X |  |  |  |  |  |
|  | UK Medical Careers Research Group |  | UK | Doctor | 16 | 8 | 1975 | Professional registry | Census, nationally representative | Paper and online link | 2347 | Paper and online link | Mail and email reminder | 1545 | 2014 | N/A | N/A | X |  |  |  |  |  |
|  | University of Auckland's Health Career Pathways Project |  | New Zealand | Nurse | 11 | 1 | 2006 | Training institutions | Single institution, not nationally representative | N/A | N/A | N/A | N/A | N/A | N/A | N/A | N/A | X |  |  |  |  |  |
|  | Park J et al. UK nurse cohort |  | UK | Nurse | 7 | 2 | 1994 | Training institutions | Single institution, not nationally representative | N/A | 10 | N/A | N/A | 7 | 1999 | N/A | N/A | X |  |  |  |  |  |
|  | Kirkpatrick, H.   and Wasfie, T.   and Laykova, A.   and Barber, K.   and Hella, J.   and Vogel, M. |  | US | Doctor | 2 | 3 | 2019 | Health facility | Single facility, not nationally representative | N/A | N/A | N/A | N/A | N/A | N/A | N/A | N/A |  |  | X | MBI, physician wellness inventory |  |  |
|  | Campbell N et al. Australia HCW cohort |  | Australia | HCW | 4 | 1 | 2020 | Training institutions | Single institution, not nationally representative | Online link or phone | N/A | Online link or phone | Financial | N/A | N/A | N/A | N/A | X |  |  |  |  |  |
|  | Petrides K and McManus I UK medical student study |  | UK | Doctor | 3 | 1 | 1981 | Training institutions | Single institution, not nationally representative | Paper | 1478 | Paper | N/A | 961 | 1986 | N/A | N/A | X |  |  |  |  |  |
|  | Ward A et al. Australia doctor cohort |  | Australia | Doctor | 2 | 1 | 1984 | Training institutions | Single institution, not nationally representative | N/A | N/A | Mail and phone | N/A | N/A | N/A | N/A | N/A | X |  |  |  |  |  |
|  | Jamieson J et al. Canada doctor cohort |  | Canada | Doctor | N/A | 2 | N/A | Training institutions | Single institution, not nationally representative | Mail | 222 | Mail | Reminder | N/A | N/A | N/A | N/A | X |  |  |  |  |  |
|  | Hogenbirk J et al. Canada doctor cohort |  | Canada | Doctor | N/A | N/A | 2005 | Training institutions | Single institution, not nationally representative | N/A | N/A | N/A | N/A | N/A | N/A | Training institution database | N/A | X |  |  |  |  |  |
|  | Schmit Jongbloed L et al. Netherland doctor cohort |  | Netherlands | Doctor | 4 | N/A | 1982 | Training institutions | 2 institutions, not nationally representative | N/A | 166 | N/A | N/A | N/A | N/A | N/A | N/A | X |  |  |  |  |  |
|  | Owen J et al. US doctor cohort |  | US | Doctor | 6 | 1 | 1990 | Training institutions | Single institution, not nationally representative | Mail | 800 | Mail | N/A | N/A | N/A | N/A | N/A | X |  |  |  |  |  |
|  | Kawamoto R et al. Japan doctor cohort |  | Japan | Doctor | 8 | 1 | 2013 | Training institutions | Single institution, not nationally representative | N/A | N/A | Email | N/A | N/A | N/A | N/A | N/A | X | Rural self-efficacy |  |  |  |  |
|  | Poole P and Shulruf B New Zealand doctor study |  | New Zealand | Doctor | 2 | 1 | 2006 | Training institutions | Single institution, not nationally representative | N/A | N/A | N/A | N/A | N/A | N/A | N/A | Student ID | X |  |  |  |  |  |
|  | McManus I et al. UK doctor cohort |  | UK | Doctor | 3 | 2 | 1991 | Training institutions | 5 institutions, not nationally representative | Mail | N/A | Mail | N/A | N/A | N/A | N/A | N/A | X | Approach to Work Questionnaire (aAWQ), Workplace Climate Questionnaire (aWCQ) | X | GHQ-12, MBI |  |  |
|  | Pfarrwaller E et al. Switzerland medical student cohort |  | Switzerland | Doctor | 2 | 3 | 2011 | Training institutions | Single institutions, not nationally representative | Paper | 290 | Paper | N/A | N/A | N/A | N/A | Student ID | X |  |  |  |  |  |
|  | Cohen-Schotanus J et al. Netherlands medical student cohort |  | Netherlands | Doctor | 2 | N/A | 1982 | Training institutions | Single institution, not nationally representative | N/A | 398 | Phone | N/A | N/A | N/A | N/A | N/A | X |  |  |  |  |  |
|  | Mader E et al. US medical student cohort |  | US | Doctor | 2 | 3 | 2010 | Training institutions | Single institution, not nationally representative | Paper | 159 | Email | Financial | 100 | 2013 | N/A | N/A | X |  |  |  |  |  |
|  | Pelletier D et al. Australia nurse cohort |  | Australia | Nurse | 5 | 3 | 1992 | Training institutions | Single institution, not nationally representative | N/A | 70 | Mail | N/A | 33 | 1998 | N/A | N/A | X |  |  |  |  |  |
| Baseline + data linkage | Nursing and Allied Health Graduate Outcomes Tracking (NAHGOT) | X | Australia | Nurse, midwife, and allied health professional | 1 | N/A | 2017 | Training institutions | Three institutions, not nationally representative | N/A | 1130 | N/A | N/A | N/A | N/A | Regulator database | Unique ID (masking student ID and regulator's ID) | X |  |  |  |  |  |
|  | Finnish Hospital Personnel | X | Finland | Doctor, nurse | 1 | N/A | 1997 | Health facility | 11 institutions, not nationally representative | Mail | 930 | N/A | N/A | N/A | N/A | Hospital employers's register | N/A | X | Harris scale, job content questinnaire, team climate inventory | X | GHQ-12 | X |  |
|  | Abrahamsen B. Norway nurse cohort |  | Norway | Nurse | 1 | N/A | 2001 | Training institutions | Four institutions, not nationally representative | Paper | 445 | N/A | N/A | N/A | N/A | Registry data (statistics norway) | N/A | X |  |  |  |  |  |
|  | Abelson J et al. US surgeon cohort |  | US | Doctor | N/A | N/A | 2007 | Professional registry | All surgery resident, national representative | N/A | N/A | N/A | N/A | N/A | N/A | Registry data | N/A | X |  |  |  |  |  |
|  | Scott I et al. Canada medical student cohort |  | Canada | Doctor | 1 | N/A | 2002 | Training institutions | Half of the eligible institutions, not nationally representative | N/A | N/A | N/A | N/A | N/A | N/A | Registry data | N/A | X |  |  |  |  |  |
|  | Hurtado D et al. US nurse study |  | US | Nurse, nurse assistant | 1 | N/A | 2016 | Health facility | Single facility, not nationally representative | Online link | 35 | N/A | N/A | N/A | N/A | Hospital employers's register | N/A | X |  |  |  |  |  |
|  | Arora V et al. US doctor study |  | US | Doctor | 3 | N/A | 2001 | Training institutions | Six institution, not nationally representative | N/A | 402 | N/A | N/A | N/A | N/A | Residency database | N/A | X |  |  |  |  |  |
|  | Chen Y et al. Taiwan nurse study |  | Taiwan | Nurse | 1 | N/A | N/A | Health facility | Three institutions, not nationally re representative | Paper | 553 | N/A | N/A | N/A | N/A | Hospital employers's register | N/A | X |  | X | ProQOL | X | SF-6 |
|  | Suzuki E et al. Japan nurse study |  | Japan | Nurse | 1 | 3 | 2003 | Health facility | 20 institutions, not nationally re representative | Paper | 1030 | N/A | N/A | N/A | N/A | Hospital employers's register | N/A | X |  | X | MBI |  |  |
|  | Symer M et al. US doctor study |  | US | Doctor | 1 | N/A | 2007 | Training institutions | All program, national representative | Mail | 870 | N/A | N/A | N/A | N/A | Professional registry | N/A | X |  |  |  |  |  |
|  | Chen W et al. Taiwan nurse study |  | Taiwan | Nurse | 1 | N/A | 2005 | Health facility | Single facility, not nationally representative | Online link | 170 | N/A | N/A | N/A | N/A | Internal | N/A | X |  | X |  |  |  |
|  | Sobral D et al. Brazil doctor study |  | Brazil | Doctor | 1 | N/A | N/A | Training institutions | Single institution, not nationally representative | N/A | N/A | N/A | N/A | N/A | N/A | N/A | N/A | X |  |  |  |  |  |
|  | Woolley T et al. Australia doctor study |  | Australia | Doctor | 1 | N/A | 2006 | Training institutions | Single institution, not nationally representative | N/A | N/A | N/A | N/A | N/A | N/A | Regulator database | N/A | X |  |  |  |  |  |
|  | DAK-Gesundheit nurse study |  | Germany | Nurse | 1 | N/A | 2011 | Insurance scheme | Single institution, not nationally representative | Mail | N/A | N/A | N/A | N/A | N/A | Insurance company record | Unique ID | X |  |  |  |  |  |
|  | Salamonson Y et al. Australia nurse study |  | Australia | Nurse | 1 | N/A | 2004 | Training institutions | Single institution, not nationally representative | N/A | 357 | N/A | N/A | N/A | N/A | Training institution database | Student ID | X |  |  |  |  |  |
|  | Steenstra I et al. Netherland HCW study |  | Netherlands | HCW | 1 | 1 | 1999 | Health facility | Single facility, not nationally representative | N/A | 822 | N/A | N/A | N/A | N/A | Hospital employers's record | N/A | X |  |  |  |  |  |
|  | Peters V et al. Netherlands nurse cohort |  | Netherlands | Nurse | 1 | N/A | 2009 | Health facility | 2 facilities, not nationally representative | Mail | 462 | N/A | N/A | N/A | N/A | Hospital employers's record | Unique ID | X | UWES-9 | X | MBI |  |  |
|  | Kristensen T et al. Denmark HCW cohort |  | Denmark | HCW | 1 | N/A | 2000 | Health facility | Subnational sample, not national representative | N/A | 2331 | N/A | N/A | N/A | N/A | Hospital employers's record | N/A | X | COPSOQ II |  |  |  |  |
|  | Sjoberg A and Sverke M Sweden nurse study |  | Sweden | Nurse | 1 | N/A | N/A | Health facility | Single institutions, not nationally representative | N/A | 535 | N/A | N/A | N/A | N/A | Hospital employers's record | N/A | X | Job involvement (Kanungo) |  |  |  |  |
|  | Chen H et al. Taiwan nurse study |  | Taiwan | Nurse | 1 | N/A | 2000 | Health facility | Single institution, not nationally representative | Mail | 308 | N/A | N/A | N/A | N/A | Hospital employers's record | N/A | X |  |  |  |  |  |
|  | Ogata Y et al. Japan nurse study |  | Japan | Nurse | 1 | 1 | 2013 | Health facility | 8 institutions, not nationally representative | Mail | 3137 | N/A | N/A | N/A | N/A | Hospital manager's register | N/A | X | PES-NWI | X | Negative acts questionnaire, K6, MBI | X |  |
| Baseline + short repeated measure | Resident Activity Tracker Evaluation (RATE) |  | US | Doctor | 1 | N/A | 2016 | Health facility | Single institution, not nationally representative | N/A | 59 | N/A | N/A | N/A | N/A | N/A | N/A | X |  | X | MBI | X | SF-6, Epworth Sleepiness Scale, International Physical Activity Questionnaire |
|  | Duan-Porter et al. US nursing cohort |  | US | Nurse | 1 | 11 | 2013 | Health facility | Single facility, not nationally representative | Email | 281 | Email | Financial | N/A | N/A | N/A | N/A | X | Copenhagen Psychosocial Questionnaire II, COPE | X | OLBI, PHQ-9 | X |  |
|  | Albert-Sabater J et al. Spain nurse cohort |  | Spain | Nurse | 1 | 3 | 2011 | Health facility | Single facility, not nationally representative | Paper | 205 | N/A | N/A | 188 | 2012 | N/A | N/A | X |  |  |  | X |  |
|  | Chang W et al. Taiwan nurse study |  | Taiwan | Nurse | 1 | 6 | 2017 | Health facility | Single facility, not nationally representative | Face-to-face interview | 198 | N/A | N/A | N/A | N/A | N/A | N/A | X |  |  |  | X |  |
|  | Hatch D et al. US nurse study |  | US | Nurse | 1 | 12 | N/A | Health facility | Single facility, not nationally representative | Online link | 402 | Online link | N/A | N/A | N/A | N/A | N/A | X | COPSOQ | X | OLBI, PHQ-9 |  |  |
|  | Frogeli E et al. Sweden nurse cogort |  | Sweden | Nurse | 1 | N/A | 2015 | Training institutions | All institutions providing training, nationaly presentative | Email | 267 | Email | N/A | 762 | 2007 | N/A | N/A | X |  | X | Stress and energy questionnaire |  |  |
|  | Jiang L et al. Canada HCW study |  | Canada | HCW | N/A | N/A | 2010 | Health facility | 9 institutions, not nationally representative | Online link | N/A | N/A | N/A | N/A | N/A | N/A | N/A | X |  |  |  |  |  |
| Baseline only | Careers in Rural Health Tracking Survey (CIRHTS) |  | Australia | Doctor, nurse, allied health professional | 1 | N/A | 2006 | Training institutions | Single institution, not nationally representative | N/A | 121 | N/A | N/A | N/A | N/A | N/A | N/A | X |  |  |  |  |  |
|  | COVID-19 HEalth caRe wOrkErS | X | 26 countries | HCW | 1 | N/A | 2020 | Mixed | Convenience sample in some countries, not national representative | N/A | 34000 | N/A | N/A | N/A | N/A | N/A | N/A | X |  | X | GHQ-12, PHQ-9, Columbia Suicide Severity Rating Scale, Brief Resilience Scale |  |  |
|  | COVID-19 Study of Healthcare and Support Personnel | X | US | HCW | 1 | 5 | 2020 | Professional association, trade union, social media | Convenience sample, not nationally representative | Online link | 467 | Email | N/A | N/A | N/A | N/A | N/A | X | OSSS-3 | X | GAD-7, PHQ-2, IES-R, BRS, OLBI | X |  |
|  | Doctors' e-Cohort (DeC) | X | Australia | Doctor | N/A | N/A | 2009 | Training institutions and professional registry | Only 1.2% but appear to be representative of age, work hours and country of training but not sex and current workplace | Online link | 1666 | N/A | N/A | N/A | N/A | N/A | N/A | X |  | X |  | X | SF-36 |
|  | Wood E et al. UK nursing cohort |  | UK | Nurse | 1 | N/A | 2019 | Professional association, training institution and social media | Not nationally representative | Online link | 86 | N/A | Prize draw | N/A | N/A | N/A | N/A | X |  | X | Short Warwick and Edinburgh Mental Well-being Scale |  |  |
|  | Darnton R et al. UK medical student cohort |  | UK | Doctor | 1 | N/A | 2020 | Training institutions | Three institutions, not nationally re representative | Online link | 483 | N/A | N/A | N/A | N/A | N/A | N/A | X |  |  |  |  |  |
|  | Grainger P and Bolan C Canada nursing student cohort |  | Canada | Nurse | 1 | N/A | N/A | Training institutions | Subnational sample, not national representative | N/A | 213 | N/A | N/A | N/A | N/A | N/A | N/A | X | nursing attitude questionnaire, nursing orientation tool |  |  |  |  |
| Data linkage | UKMED | X | UK | Doctor | N/A | N/A | N/A | N/A | N/A | N/A | N/A | N/A | N/A | N/A | N/A | Multiple dataset | Multiple ID (UCAS, Medical school and GMC ID) | X |  |  |  |  |  |
|  | Shelker W et al. New Zealand medical student cohort |  | New Zealand | Doctor | N/A | N/A | N/A | N/A | N/A | N/A | N/A | N/A | N/A | N/A | N/A | Registry data | N/A | X |  |  |  |  |  |
|  | Lie J et al. Norway nurse cohort |  | Norway | Nurse | N/A | N/A | N/A | N/A | All Norweign nurse, nationally representative | N/A | N/A | N/A | N/A | N/A | N/A | Disease registry (Cancer) | Government ID or name and DOB | X |  |  |  | X |  |
|  | Maltezou H et al. Greece HCW study |  | Greece | HCW | N/A | N/A | N/A | Health facility | Single facility, not nationally representative | N/A | N/A | N/A | N/A | N/A | N/A | Internal | N/A | X |  |  |  | X |  |
|  | Andreassen L et al. Norway doctor study |  | Norway | Doctor | N/A | N/A | N/A | Government authority | All Norway physician, nationally representative | N/A | N/A | N/A | N/A | N/A | N/A | Registry data (statistics norway) | N/A | X |  |  |  |  |  |
|  | Woloschuk W et al. Canada doctor study |  | Canada | Doctor | N/A | N/A | 1996 | Training institutions | Single institution, not nationally representative | N/A | N/A | N/A | N/A | N/A | N/A | Registry data | N/A | X |  |  |  |  |  |
|  | O'Sullivan B et al. Australia doctor study |  | Australia | Doctor | N/A | N/A | N/A | Training institutions | Single institution, not nationally representative | N/A | N/A | N/A | N/A | N/A | N/A | Internal and to research database | Student ID | X |  |  |  |  |  |
|  | Randolph P US nurse study |  | US | Nurse | 1 | N/A | N/A | Training institutions | Single institution, not nationally representative | N/A | N/A | N/A | N/A | N/A | N/A | Research database (Centre for health information and research at Arizona state university) | N/A | X |  |  |  |  |  |
|  | Fisher D et al. US HCW cohort |  | US | HCW | N/A | N/A | N/A | Health facility | Subnational sample, not national representative | N/A | N/A | N/A | N/A | N/A | N/A | Internal | N/A | X |  |  |  |  |  |
|  | Jacques L and Gray G US doctor study |  | US | Doctor | N/A | N/A | N/A | Training institutions | Single institution, not nationally representative | N/A | N/A | N/A | N/A | N/A | N/A | Residency database | N/A | X |  |  |  |  |  |
|  | Spetz J et al. US nurse study |  | US | Nurse | N/A | N/A | N/A | Training institutions | Subnational sample, not national representative | N/A | N/A | N/A | N/A | N/A | N/A | Government employment database | Social security number | X |  |  |  |  |  |
|  | Bennett M et al. US nursing student study |  | US | Nurse | 5 | 6 | 2012 | Training institutions | Single institution, not nationally representative | N/A | N/A | N/A | N/A | N/A | N/A | Training institution database | Student ID | X |  |  |  |  |  |
|  | Pyne Y and Ben-Shlomo Y UK doctor cohort |  | UK | Doctor | N/A | N/A | N/A | Professional registry | Nationally representative | N/A | N/A | N/A | N/A | N/A | N/A | Internal | N/A | X |  |  |  |  |  |
|  | Studerus L et al. Switzerland doctor cohort |  | Switzerland | Doctor | N/A | N/A | N/A | Training institutions | Nationally representative | N/A | N/A | N/A | N/A | N/A | N/A | Training institution database | N/A | X |  |  |  |  |  |
|  | Virtanen M et al. Finland HCW study |  | Finland | HCW | 1 | 2000 | N/A | Health facility | 5 institutions, not nationally representative | N/A | N/A | N/A | N/A | N/A | N/A | Employer registry and insurance database | N/A | X |  | X |  |  |  |
|  | Khalafallah A et al. US doctor study |  | US | Doctor | N/A | N/A | N/A | Professional registry | All neurosurgeon resident, national representative | N/A | N/A | N/A | N/A | N/A | N/A | Web-based search | N/A | X |  |  |  |  |  |
|  | Buchbinder S et al. US doctor cohort |  | US | Doctor | 1 | 1 | 1987 | Professional association | Nonfederally employed, not nationally representative | N/A | N/A | N/A | N/A | N/A | N/A | Research database (Robert Wood Johnson Foundation) | N/A | X |  |  |  |  |  |
|  | Shires L et al. Australia doctor study |  | Australia | Doctor | N/A | N/A | N/A | Training institutions | Single institution, not nationally representative | N/A | N/A | N/A | N/A | N/A | N/A | Regulator database | Name | X |  |  |  |  |  |
|  | Cole D et al. Canada HCW study |  | Canada | HCW | N/A | N/A | N/A | Health facility | Subnational sample, not national representative | N/A | N/A | N/A | N/A | N/A | N/A | Employer registry, health record, insurance claim, census and administrative database | Name, DOB and social security number | X |  | X |  | X |  |
|  | Mowat S et al. Canda doctor study |  | Canada | Doctor | N/A | N/A | N/A | Training institutions | Single institution, not nationally representative | N/A | N/A | Email | N/A | N/A | N/A | Professional registry | N/A | X |  |  |  |  |  |
|  | Pagaiya N et al. Thailand doctor study |  | Thailand | Doctor | N/A | N/A | N/A | N/A | All Thailand doctor trainee, nationally representative | N/A | N/A | N/A | N/A | N/A | N/A | Government website | N/A | X |  |  |  |  |  |
|  | Meyers P et al. US doctor study |  | US | Doctor | N/A | N/A | N/A | N/A | All family resident, nationally representative | N/A | N/A | N/A | N/A | N/A | N/A | Residency database | N/A | X |  |  |  |  |  |
|  | Jaakkimainen R et al. Canda doctor cohort |  | Canada | Doctor | N/A | N/A | 1993 | Training institutions | Single institution, not nationally representative | N/A | N/A | N/A | N/A | N/A | N/A | Government employment database | N/A | X |  |  |  |  |  |
|  | Rauhala A et al. Finland nurse study |  | Finland | Nurse | N/A | N/A | 2004 | Health facility | 5 institutions, not nationally representative | N/A | N/A | N/A | N/A | N/A | N/A | Hospital employers's record | N/A | X |  |  |  |  |  |
| Repeated survey | American Academy of PAs (AAPA) student and census survey | X | US | PA | N/A | N/A | 2000 | Professional association | Census, nationally representative | Email and mail | 3721 | Email and mail | N/A | N/A | N/A | N/A | Membership ID | X |  |  |  |  |  |
|  | Carver College of Medicine Specialty Choice survey |  | US | Doctor | 3 | 3 | 2013 | Training institutions | Single institution, not nationally representative | N/A | N/A | N/A | N/A | N/A | N/A | N/A | N/A | X |  |  |  |  |  |
|  | Japan Survey of Physicians, Dentists, and Pharmacists | X | Japan | Doctor | N/A | N/A | 1980 | Government authority | Census, nationally representative | N/A | 155530 | N/A | Government requirement | N/A | N/A | Internal | Unique ID (masking registration ID) | X |  |  |  |  |  |
|  | North Carolina Health Professions Data System for Registered Nurses |  | US | Nurse | N/A | N/A | 1984 | Professional registry | Subnational sample, not national representative | N/A | 3384 | N/A | N/A | 2418 | 2003 | N/A | N/A | X |  |  |  |  |  |
|  | Frisch, L. et al. KUSM-W family resident survey |  | US | Doctor | 1 | 4 | 1992 | Training institutions | Single facility, not nationally representative | Mail | N/A | Mail | Mail reminder | N/A | N/A | N/A | N/A | X |  |  |  |  |  |
|  | Fang Y et al. China nurse cohort |  | China | Nurse | 1 | 3 | 2020 | Health facility | Single facility, not nationally representative | Online link | 127 | Online link | N/A | 127 | 2021 | N/A | N/A | X |  | X | Nursing stressor scale |  |  |
|  | Mason S et al. UK doctor cohort |  | UK | Doctor | 1 | 3 | 2010 | Health facility | Nine deanaries out of 14, not national representative | Online link | 217 | Online link | N/A | 108 | 2011 | N/A | N/A | X | Job satisfaction scale | X |  |  |  |
|  | Magnavita N et al Italy intensive care physician cohort |  | Italy | Doctor | 1 | 2 | 2020 | Health facility | Single institution, not nationally representative | Online link | 154 | Email | N/A | 120 | 2021 | N/A | N/A | X | Colquitt questionnaire | X | Siegrist effort/reward imbalance model, GADS | X |  |
|  | Agarwal N et al. US doctor cohort |  | US | Doctor | N/A | N/A | 2005 | Professional association, training institution and social media | All neurosurgeon resident, national representative | N/A | N/A | N/A | N/A | N/A | N/A | Internal | N/A | X |  |  |  |  |  |
|  | Lin Y et al. Taiwan nurse cohort |  | Taiwan | Nurse | 1 | 6 | 2011 | Health facility | Single institution, not nationally representative | N/A | 218 | N/A | N/A | N/A | N/A | N/A | N/A | X |  | X | Beck Anxiety Inventory, nurse Stress Checklist |  |  |
|  | Association of American Medical Colleges Questionnaire | X | US | Doctor | N/A | N/A | N/A | Training institutions | All resident, national representative | Online link | N/A | Online link | N/A | N/A | N/A | Internal | Unique ID | X |  | X | OBI, PSS |  |  |
|  | McCann T et al. Australia nursing student cohort |  | Australia | Nurse | 1 | 2 | 2005 | Training institutions | Single institution, not nationally representative | Paper | 90 | Paper | N/A | 96 | 2007 | N/A | N/A | X |  |  |  |  |  |
|  | Kumar S et al. New Zealand psychiatrists survey |  | New Zealand | Doctor | 1 | 1 | 2005 | Professional registry | All psychiatrists, nationally representative | Mail | 239 | Paper | Mail reminder | N/A | N/A | N/A | Unique ID | X | Job diagnostic survey | X | MBI |  |  |
|  | Willard-Grace R et al. US doctor survey |  | US | HCW | N/A | N/A | 2013 | Health facility | Subnational sample, not national representative | N/A | 740 | N/A | N/A | N/A | N/A | N/A | N/A | X |  | X | MBI |  |  |
|  | Stevens J et al. Australia Nurse survey |  | Australia | Nurse | 1 | 2 | 2007 | Training institutions | Single institution, not nationally representative | N/A | 203 | N/A | N/A | 160 | 2009 | N/A | N/A | X |  |  |  |  |  |
|  | Alameddine M et al. Canada nurse analysis |  | Canada | Nurse | N/A | N/A | N/A | N/A | Subnational sample, not national representative | N/A | N/A | N/A | N/A | N/A | N/A | Internal | Registration ID | X |  |  |  |  |  |
|  | Martinez K et al. US doctor cohort |  | US | Doctor | 1 | 1 | 2013 | Health facility | Single facility, not nationally representative | N/A | 1220 | Email | Approaching department chiar | 149 | 2019 | N/A | N/A |  |  | X | MBI |  |  |
|  | Annual Internal Medicine In-Training Examination survey (IM-ITE) | X | US | Doctor | N/A | N/A | N/A | N/A | All internal medicine resident, national representative | N/A | N/A | N/A | N/A | N/A | N/A | Internal | N/A | X |  |  |  |  |  |
|  | Matsumoto M et al. Japan doctor cohort |  | Japan | Doctor | 5 | N/A | 2014 | Training institutions | Single institution, not nationally representative | N/A | N/A | N/A | N/A | N/A | N/A | Internal and to government database | Unique ID | X |  |  |  |  |  |
|  | Shopen N et al. Israel doctor cohort |  | Israel | Doctor | 1 | 1 | 2020 | Health facility | 15 facilities, not nationally representative | N/A | 84 | N/A | N/A | 39 | 2021 | N/A | N/A | X | Work and meaning inventory | X | MBI |  |  |
|  | Playford D et al. Australia GP cohort |  | Australia | Doctor | 1 | 10 | 2008 | Government authority | Subnational sample, not national representative | N/A | 665 | N/A | N/A | N/A | N/A | N/A | N/A | X |  |  |  |  |  |
|  | Do D et al. Bangladesh HCW study |  | Bangladesh | HCW | 1 | N/A | 2019 | Health facility | All secondary and teritary facilities, nationally representative | N/A | N/A | N/A | N/A | N/A | N/A | N/A | N/A | X |  |  |  |  |  |
|  | Lundgren S et al. Sweden nurse study |  | Sweden | Nurse | 1 | 2 | N/A | Health facility | Single facility, not nationally representative | Paper | 12 | Paper | N/A | N/A | N/A | N/A | N/A | X |  |  |  |  |  |
|  | Kowalenko T et al. US HCW cohort |  | US | HCW | N/A | N/A | N/A | Health facility | 6 facilities, not nationally representative | Online link | N/A | Online link | Financial | N/A | N/A | N/A | N/A | X |  |  |  |  |  |
