# Supplementary material for: Longitudinal tracking of healthcare professionals: a methodological scoping review
Source: BMC Med Res Methodol. 2025 Apr 1;25:83. doi: 10.1186/s12874-025-02533-1 (PMC11959867; doi:10.1186/s12874-025-02533-1)
Supplement: Supplementary file 3 — Additional file 3: Microsoft word document (.doc); Detail on cohort and multiple cohort studies. [file 12874_2025_2533_MOESM3_ESM.docx]

**Additional file 3. Methodological details on cohort and multiple cohort studies**

**Table 1. Characteristics and detail**

| **Characteristics** | **Detail** |
| --- | --- |
| Study country or territory | - Most commonly US (n=41), UK (n=19), Australia (n=17), Netherlands (n=14) and Canada (n=12) - 14 cohorts conducted in LMICs: China (n=8), South Africa (n=2), Thailand (n=2), Brazil (n=1), Ethiopia (n=1), - 5 studies included more than one countries |
| Study population | - Doctors (n=75, out of which 19 focused on only qualified, 18 focused on only students, others focused on a mix of student, intern, qualified) - Nurses (n=82, out of which 53 focused on only qualified, 12 focused on only students, others similarly focused on a mix of student, intern, qualified) - General HCW (N=18) - Others such as nurse assistants, midwives (n=5) |
| Rounds of “cohort” and “followup” | - Most only have 1 cohort (n=152) - 95 studies only followed up once, 34 followed up twice, 21 followed up three times, others more than 3 times |
| Baseline recruitment | - 89 studies recruited from training institutions - 62 from health facilities - 23 from professional associations, professional registries or trade unions - Others from government authority or social media - 28 studies seems to be nationally representative (very hard to judge!) |
| Baseline platform | - 62 studies did not report their baseline data collection platform - 36 used papers, 32 used mails - 15 used emails, 13 used online link - 3 used face-to-face interview - Others used a mix of paper, email, phone |
| Followup platform | - 55 did not report their followup data collection platform - 36 used mail, 24 used papers - 22 used email, 10 used online link - 2 used face-to-face interview - 1 study used SMS and 1 used phone - Others used a mix of paper, email, SMS, phone, online link |
| Retention strategy | - 140 studies did not report any strategy - 12 used financial incentives, 19 used different reminders |
| Data linkage | - 147 studies did not report how they linked - 23 studies reported the use of an unique ID, 3 studies simply used their names - Other strategies include national ID, social security number, student ID, birth date and postcode |
| Linkage to other database | - 14 studies reported linkage to other dataset such as hospital employer’s record, regulator database, etc. |
| Funding source | - Around half did not report their funding information or received no funding - 61 received government funding (e.g. research funding), 10 from research institutions, 4 from local institution funding, 4 ODA funding, 3 from philanthropy, 3 from professional association, 3 from other association, 2 from corporate (insurance), 1 from registry, 11 from mixed sources |

***Table 2. Retention rate between baseline and latest available follow-up***

Note: Each dot represents one study. Y-axis is the retention proportion, and X-axis is the year between baseline and follow-up (note that if the follow-up is conducted within 12 months of the initial follow-up, it was counted as zero)
